# Solar-driven methanogenesis with ultrahigh selectivity by turning down H_2_ production at biotic-abiotic interface

**DOI:** 10.1038/s41467-022-34423-1

**Published:** 2022-11-03

**Authors:** Jie Ye, Chao Wang, Chao Gao, Tao Fu, Chaohui Yang, Guoping Ren, Jian Lü, Shungui Zhou, Yujie Xiong

**Affiliations:** 1 Fujian Provincial Key Laboratory of Soil Environmental Health and Regulation, College of Resources and Environment, Fujian Agriculture and Forestry University, Fuzhou, 350002 China; 2 School of Chemistry and Materials Science, University of Science and Technology of China, Hefei, 230026 China

**Keywords:** Photocatalysis, Biocatalysis, Photocatalysis, Characterization and analytical techniques, Bacterial techniques and applications

## Abstract

Integration of methanogens with semiconductors is an effective approach to sustainable solar-driven methanogenesis. However, the H_2_ production rate by semiconductors largely exceeds that of methanogen metabolism, resulting in abundant H_2_ as side product. Here, we report that binary metallic active sites (namely, NiCu alloys) are incorporated into the interface between CdS semiconductors and *Methanosarcina barkeri*. The self-assembled *Methanosarcina barkeri*-NiCu@CdS exhibits nearly 100% CH_4_ selectivity with a quantum yield of 12.41 ± 0.16% under light illumination, which not only exceeds the reported biotic-abiotic hybrid systems but also is superior to most photocatalytic systems. Further investigation reveal that the Ni-Cu-Cu hollow sites in NiCu alloys can directly supply hydrogen atoms and electrons through photocatalysis to the *Methanosarcina barkeri* for methanogenesis via both extracellular and intracellular hydrogen cycles, effectively turning down the H_2_ production. This work provides important insights into the biotic-abiotic hybrid interface, and offers an avenue for engineering the methanogenesis process.

## Abstract (web summary)

While the combination of synthetic and biological systems offers an appealing strategy for solar-to-fuel conversion, such hybrid systems typically suffer from low selectivity. Here, authors integrate a bimetallic alloy with a CdS-containing methanogen for selective CO_2_ reduction to methane.

## Introduction

Effective conversion of carbon dioxide (CO_2_) to value-added low-carbon biofuels is an important strategy for alleviating energy shortages and offsetting global carbon emissions via industrial CO_2_ capture that displaces fossil fuel or natural gas use^1^. It is estimated that over 0.5 gigatonnes of CO_2_ can be utilized annually via the CO_2_-to-fuel pathway and the value increases every year, which significantly improves the CO_2_ emission-reduction potential globally with the significant technological and societal importance^2,3^. Elegant advances have achieved for CO_2_ reduction via biocatalytic, electrocatalytic and photocatalytic approaches; however, these processes still face formidable challenges, mainly related to the limited solubility of electron carriers in water, low catalyst activity and product selectivity, and high energy barriers for reactions^3–5^. The limitations become more prominent when methane (CH_4_), which has a high calorific value of 890 kJ mol^−1^ and can be integrated into the existing energy infrastructure, is chosen as the target product^6^. The conversion of CO_2_ to CH_4_ is a kinetically complex and energetically intensive process that involves multiple proton-coupled electron transfer steps and requires to finely tune activation energy for promoting the forward reaction^7^. Solar-driven CO_2_-to-CH_4_ conversion is an ideal approach in terms of energy input, and yet is largely limited by unsatisfactory reaction activity and product selectivity.

Biotic-abiotic hybrid systems, which have been developed by integrating biological whole-cell bacteria with man-made semiconductor materials in recent years, offer new opportunities for the solar-driven CO_2_-to-CH_4_ conversion by taking advantage of both photocatalysis and biocatalysis^8–13^. In a typical hybrid system, methanogens can sustainably harness the reducing equivalents from biocompatible semiconductors with broadband light harvesting efficiency to realize effective solar-driven CO_2_-to-CH_4_ conversion^12^. In most cases, the electron transfer and utilization across the biotic-abiotic interface govern the methanogenesis performance^14^. Nevertheless, it is worth pointing out that direct interspecies electron transfer (DIET) is not the exclusive process for supplying reducing equivalents. H_2_ is a preferred reducing equivalent that dominates the electron transport chain in biotic-abiotic hybrid systems at large timescales and conserves substantially more energy in CO_2_-to-CH_4_ conversion^15,16^. Photocatalysis by the semiconductor materials can generate H_2_ from water medium, providing the H_2_ reducing equivalents for methanogens. However, high energy conservation can only be achieved at the compensation of high H_2_ threshold concentration, particularly for methanogens with cytochromes such as *Methanosarcina barkeri* (*M. b*)^17^. The metabolism of methanogens becomes significantly slower near the H_2_ threshold concentration^18^, whose rate would be substantially lower than that of H_2_ production from photocatalysis. This circumstance inevitably leaves abundant H_2_ in the system, resulting in relatively low CH_4_/H_2_ selectivity (typically about 50%)^12^. To achieve ultrahigh product selectivity for CH_4_, it is imperative to develop an approach by engineering the biotic-abiotic interface that can harness the hydrogen atoms from photocatalytic reduction to avoid H_2_ production and directly transfer them to methanogens for successive biocatalytic reactions.

**Fig. 1:**
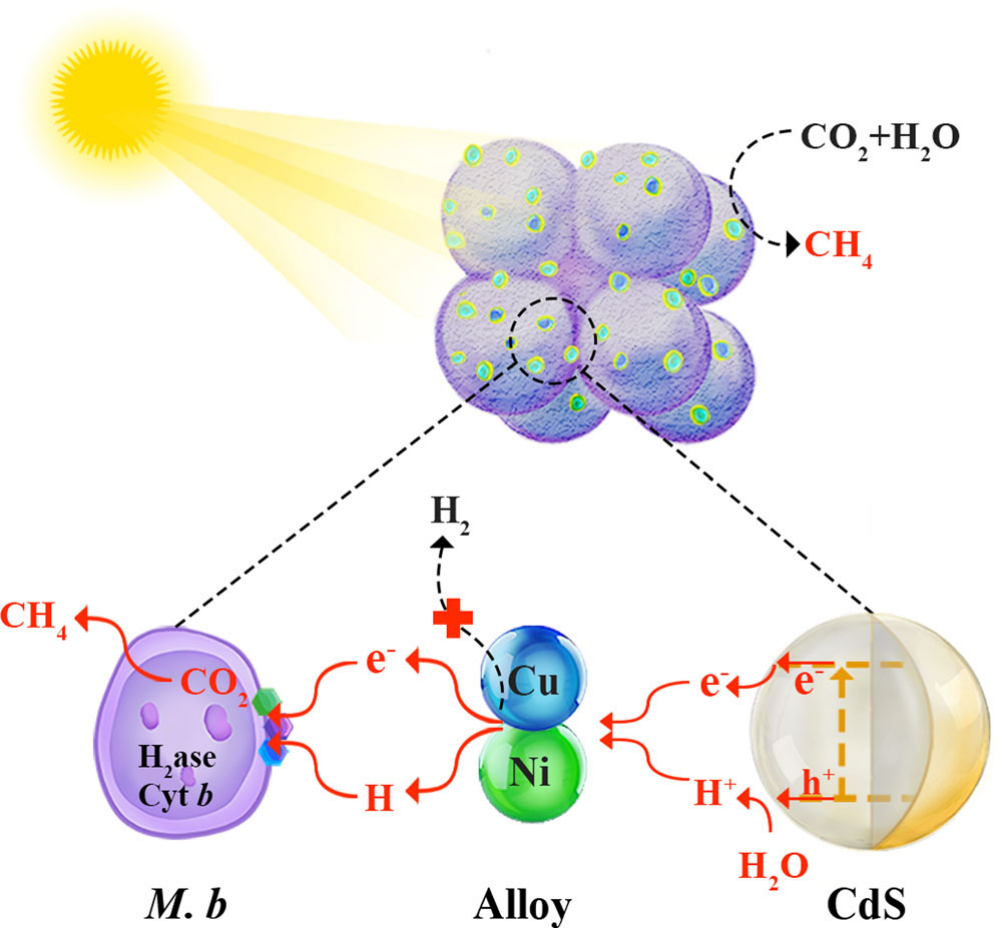
Schematic illustration for the approach to solar-driven methanogenesis with *M. b*-NiCu@CdS. In other words, the produced protons (H^+^) and photoexcited electrons (e^−^) with CdS under light illumination can transfer to the NiCu alloy, and then be harvested by *M. b* for efficient CO_2_-to-CH_4_ conversion via turning down the H_2_ production.

Here, we report the use of nickel-copper (NiCu) alloys as binary active sites for incorporation into the interface between *M. b* and cadmium sulfide (CdS) nanoparticles, designing an *M. b*-NiCu@CdS biotic-abiotic hybrid system (Fig. 1) to address this challenge. Ni and Cu are chosen for the interface engineering owing to their suitable work functions and binding strength for H atoms^19–21^. The well-designed hybrid system demonstrates the dramatically enhanced hydrogen and electron flow from semiconductor to binary active sites and finally to archaea, coupled with CO_2_ for methanogenesis. As a proof of concept, *M. b*-NiCu@CdS achieves nearly 100% CH_4_ selectivity with a rate of 79.38 ± 2.83 μmol g_cat_^−1^ h^−1^ and a quantum yield of 12.41  ± 0.16%. This work provides important insights into the design of biotic-abiotic interface for harnessing element and electron flow in hybrid photocatalytic systems, and opens an avenue for achieving sustainable and scalable conversion of CO_2_ to biofuels with ultrahigh product selectivity.

## Results

### System design and characterization

**Fig. 2:**
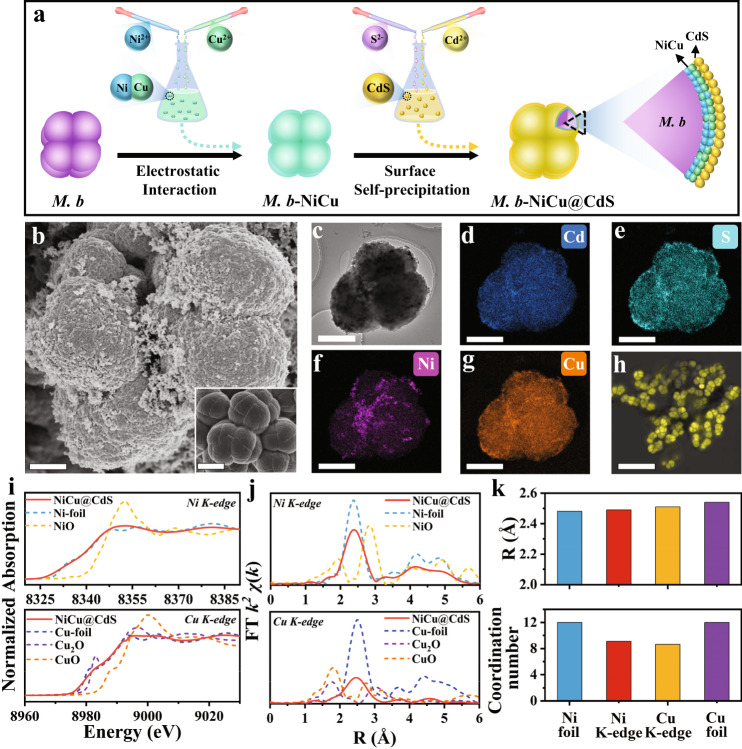
Structural characterization of *M. b*-NiCu@CdS. **a** Schematic illustration for the synthesis of *M. b*-NiCu@CdS. **b** SEM images of *M. b*-NiCu@CdS (the inset image shows bare *M. b*). **c**−**g** TEM image of *M. b*-NiCu@CdS (**c**) and the corresponding EDS mapping of Cd (**d**), S (**e**), Ni (**f**) and Cu (**g**). **h** CLSM image of *M. b*-NiCu@CdS. **i**−**j** *K*-edge XANES spectra of Ni, Cu and the references (i) and the corresponding Fourier transforms of k^2^-weighted EXAFS spectra (**j**). **k** Average bond lengths and coordination numbers in the first coordination shells of Ni and Cu atoms obtained by EXAFS spectral curve fitting. Scale bars: 1 μm in **b**, 2 μm in the insert image, 500 nm in **c**–**g**, and 10 μm in **h**. Source data are provided as a Source Data file.

The NiCu alloys are synthesized by the galvanic replacement reaction of corresponding metal ions. X-ray diffraction (XRD) patterns of NiCu alloys with different compositions show the typical face-centred cubic (fcc) crystal phase with a similar diffraction pattern between the standard Ni (JCPDS # 04-0850) and Cu peaks (JCPDS # 04-0836) (Supplementary Fig. 1a), consistent with the previously reported NiCu alloys^20^. For the optimized Ni_2_Cu_8_ (demonstrated in the following part), high-resolution transmission electron microscopy (HRTEM) image shows an interplanar distance of 0.210 nm, which matches well with the *d*-spacing value of the metallic NiCu(111) plane. The selected area electron diffraction (SAED) pattern identifies the monocrystalline nature of as-prepared Ni_2_Cu_8_, demonstrating that no any new phases are formed (Supplementary Fig. 1b). The energy-dispersive X-ray spectroscopy (EDS) mapping images also show the relatively homogeneous distribution of introduced copper atoms (Supplementary Fig. 1c–f). These results can be attributed to the similar atom radius and electronegativity of Ni and Cu that prompt the formation of alloy^22^. The formed NiCu alloys were then bound to *M. b* via electrostatic interactions based on zeta potential (Supplementary Fig. 2), followed by further integration with CdS via surface self-precipitation (Fig. 2a). The designed *M. b*-(50%Ni_2_Cu_8_)@CdS with 0.5 wt% cysteine (Cys) is chosen as the main model for biotic-abiotic hybrid systems (denoted as *M. b*-NiCu@CdS), given its highest CH_4_ yield among various composition configurations (Supplementary Fig. 3 and Supplementary Fig. 4). The combination of scanning electron microscopy (SEM, Fig. 2b), TEM (Fig. 2c), EDS mapping (Fig. 2d−g) and X-ray photoelectron spectroscopy (XPS, Supplementary Fig. 5) demonstrates the deposition of smaller nanoparticles composed of Cd, S, Ni and Cu for the encapsulation of *M. b*. Confocal laser scanning microscopy (CLSM) further confirms that the location of CdS nanoparticles, indicated by bright yellow fluorescence (a typical colour of metal chalcogenides), perfectly matches the shape of *M. b* (Fig. 2h). Further analysis reveals that the Ni/Cu ratios in the resulted biotic-abiotic hybrid system are close to the theoretical values (Supplementary Table 1), and the deposited nanoparticles are crystalline CdS and NiCu alloys (Supplementary Fig. 6).

To gain more information for interfacial binary active sites, the atomic structure of NiCu in the hybrid system is further examined by Ni and Cu *K*-edge X-ray absorption near edge structure (XANES) spectroscopy. The high similarity of the measured spectra to Ni and Cu foil references, which however greatly differ from those of NiO, CuO and Cu_2_O references, indicates the metallic nature of the NiCu alloy (Fig. 2i). The radial structure function obtained by the Fourier transform (FT) of extended X-ray absorption fine-structure (EXAFS) spectra (Fig. 2j) shows profound peaks located at ~2.3 Å and ~2.4 Å, associated with Ni−Ni and Cu−Cu bonds, respectively. In particular, the intensities of both peaks are lowered along with the coordination numbers reduced from Ni (~12.00) to NiCu (~9.08) and from Cu (~12.00) to NiCu (~8.63), representing the formation of Ni−Cu bonds upon alloying Ni and Cu. This argument is supported by the length of the formed Ni−Ni/Cu bonds between those of typical Ni−Ni and Cu−Cu bonds (Fig. 2k, Supplementary Fig. 7, and Supplementary Table 2). As such, the formation of Ni−Ni/Cu bonds results in unique binary active sites at the biotic-abiotic interface, which can improve the capability of CO_2_ adsorption (Supplementary Fig. 8). While the CO_2_ adsorption takes place over the catalysts via either O− or C−M interaction (M represents the metal active site), bimetallic atomic sites may offer both O−M and C−M interaction to enhance CO_2_ adsorption^23^. As a result, compared with CdS (*E*_ads_ = −0.16 eV), a lower CO_2_ adsorption energy (*E*_ads_ = −0.61 eV) with NiCu@CdS is observed (Supplementary Fig. 9), indicating a stronger adsorption capacity of CO_2_ on the Ni−Ni/Cu sites. Similar results were reported previously^24^, in which the paired Cu-Pd sites in Pd_7_Cu_1_ alloys were demonstrated to enhance CO_2_ adsorption and activation.

### Light-driven methanogenesis performance

**Fig. 3:**
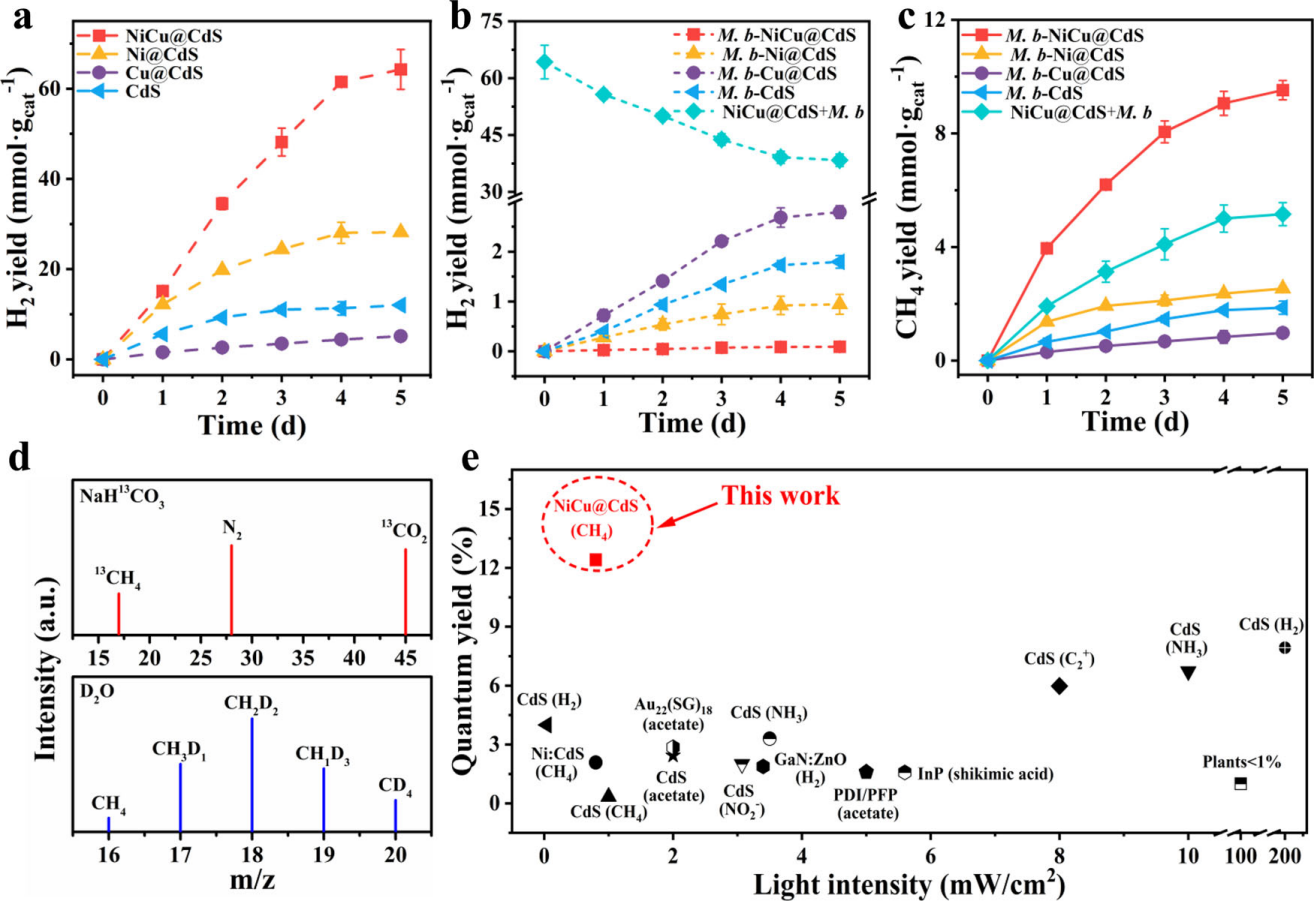
Light-driven methanogenesis performance. **a**, **b** H_2_ yields by various *M. b*-absent CdS-based materials (**a**) and biotic-abiotic *M. b*-CdS-based hybrid systems (**b**). **c** CH_4_ yields in various biotic-abiotic hybrid systems. **d** Mass spectrometric analysis of headspace gas with NaH^13^CO_3_ and D_2_O as carbon source and water medium, respectively, using *M. b*-NiCu@CdS as catalyst. **e** Comparison of the quantum yields of *M. b*-NiCu@CdS and the reported biotic-abiotic hybrid systems. All the measurements in **a**−**d** are performed in 50 mL of sterilized autotrophic medium with CO_2_ as the sole carbon source under irradiation of 395 ± 5 nm LEDs (0.8 ± 0.1 mW cm^−2^). Data are presented as mean values ± standard deviation derived from *n* = 3 independent experiments. Source data are provided as a Source Data file.

To assess the validity of our design, we first examine the essentially key side reaction—H_2_ production in 50 mL of sterilized autotrophic medium with CO_2_ as the sole carbon source under irradiation of 395 ± 5 nm LEDs (0.8 ± 0.1 mW cm^−2^). The H_2_ production of different CdS-based materials without *M. b* is first investigated (Fig. 3a). After 5 days of irradiation, the H_2_ yield by NiCu@CdS, originating from photocatalytic reduction, reaches 64.26 ± 4.42 mmol g_cat_^−1^, which is 2.28-, 12.50- and 5.35-fold higher than that of Ni@CdS, Cu@CdS and CdS, respectively. This indicates that the synergistic cooperation of Ni and Cu metallic sites contributed to the enhanced photocatalytic H_2_ production. Among the CdS-based materials without *M. b*, Cu@CdS exhibits the lowest H_2_ yield because copper ions (Cu^2+^) can be easily generated in the Cu@CdS system via a Fenton-type reaction (2H_2_O + 2 h^+^ → H_2_O_2_ + 2H^+^, Cu^0^ + h^+^ → Cu^+^, Cu^+^ + H_2_O_2_ → Cu^2+^ + OH^−^ + ∙ OH). Then the formed Cu^2+^ may react with the sulfhydryls in cysteine (RSH) (2Cu^2+^ + 2RSH → 2Cu^+^ + RSSR + 2H^+^), and reduce the function of cysteine as a sacrificial reducing agent^25^. In addition, Cu^2+^ can be transformed to CuS (*K*_sp_ of CdS: 8.0 × 10^−27^, *K*_sp_ of CuS: 1.27 × 10^−36^)^26,27^, which has been confirmed by XPS and XRD (Supplementary Fig. 10). This feature for Cu@CdS reduces the efficiency of generating and transferring photoexcited electrons, significantly limiting the photocatalytic H_2_ production. As *M. b* is added into the system of NiCu@CdS without direct interaction of *M. b* with NiCu (namely, NiCu@CdS+*M. b*, similarly to planktonic cells), we observe an obviously decrease in H_2_ concentration to 38.36 ± 1.63 mmol g_cat_^−1^. This demonstrates that the produced H_2_ can indeed be consumed by *M. b* for CO_2_ reduction; however, apparently the H_2_ cannot be depleted by the metabolism of *M. b* due to the H_2_ threshold concentration for *M. b*. As a result, the H_2_ utilization efficiency is only 40.13% resulting in a low CH_4_ selectivity of 11.85% (Supplementary Table 3). In sharp contrast, dramatically lower H_2_ concentration can be detected in the biotic-abiotic hybrid systems, particularly with *M. b*-NiCu@CdS (Fig. 3b). This suggests that the intimate contact between *M. b* and NiCu@CdS through our interface engineering can effectively transfer the hydrogen atoms, formed through photocatalytic reduction, to *M. b* for methanogenesis, effectively suppressing H_2_ production as a side reaction. Moreover, almost no CO is detected with *M. b*-NiCu@CdS, most likely as CO can serve as not only an effective quencher for reactive species but also a carbon source for *M. b*^28^.

Leveraging the efficient hydrogen transfer, an impressive CH_4_ yield of >9.50 mmol g_cat_^−1^ is obtained with *M. b*-NiCu@CdS, along with nearly 100% CH_4_ selectivity (99.04%, Fig. 3c). The product selectivity and production rate (79.38 ± 2.83 μmol g_cat_^−1^ h^−1^) not only exceed the reported biotic-abiotic hybrid systems (Supplementary Table 4), but also are superior to most photocatalytic systems (Supplementary Table 5). In contrast, the absence of NiCu alloy leads to an 89.3% decrease in CH_4_ yield under the same conditions (Fig. 3c). To further evaluate the role of hydrogen in methanogenesis with *M. b*-NiCu@CdS, 1,4-diphenyl-butadiyne (DPB) is decorated on the NiCu@CdS surface as a hydrogen scavenger. An 83.3% reduction in CH_4_ yield is observed in the presence of DPB, demonstrating the important role of hydrogen in the methanogenesis of *M. b*-NiCu@CdS.

Previous research indicated that cysteine could be biologically assimilated in the CdS-based hybrid systems as potential carbon and energy sources;^29,30^ however, our growth experiment demonstrates that cysteine is not growth-supportive for *M. b* with the insignificant change (*p* > 0.05) of optical density (OD_600_) and CH_4_ yield (Supplementary Fig. 11). The ^12^C nuclear magnetic resonance (NMR) spectra, in which no characteristic peaks for the metabolites of cysteine such as pyruvate and acetate can be detected (Supplementary Fig. 12), demonstrate that cysteine does not serve as a carbon source. Isotopic labelling experiments are further conducted to confirm the CH_4_ source in the *M. b*-NiCu@CdS hybrid system with 0.5 wt.% cysteine as a sacrificial agent. In addition to the ubiquitous N_2_ (*m/z* = 28) and ^13^CO_2_ (*m/z* = 45), a characteristic peak of ^13^CH_4_ (*m*/*z* = 17) is found with ^13^C-labled NaHCO_3_ as the sole carbon source, whereas such a peak of ^13^CH_4_ (*m*/*z* = 17) disappears when ^13^C-labled NaHCO_3_ is replaced by ^13^C-labled cysteine (Fig. 3d, Supplementary Fig. 13), demonstrating that the produced CH_4_ is derived from CO_2_ reduction. Moreover, an excellent methanogenesis performance is also achieved by *M. b*-NiCu@CdS hybrid system after the replacement of cysteine with potassium iodide (KI) (Supplementary Fig. 14). A CH_4_ yield of >80% (based on the initial KI concentration) provides the indirect evidence that cysteine only acts as a reducing agent for methanogenesis, rather than an effective carbon source for *M. b*. Furthermore, the formation of CH_3_D (21.79%), CH_2_D_2_ (30.06%), CHD_3_ (20.96%) and CD_4_ (15.21%) with D_2_O as a solvent demonstrates the participation of water molecules, in addition to free protons, during methanogenesis with *M. b*-NiCu@CdS. A prominent quantum yield of 12.41 ± 0.16% is achieved during CO_2_-to-CH_4_ conversion with *M. b*-NiCu@CdS (Supplementary Fig. 15). It is worth noting that such a value is substantially higher than the year-long averages determined for crops and forests (0.2– 0.4%)^31^ and those of the reported biotic-abiotic hybrid systems, even for more kinetically favourable two-electron reactions such as H^+^-to-H_2_ and NO_3_^–^ to-NO_2_^-^ reduction (Fig. 3e and Supplementary Table 4). Meanwhile, the energy efficiency of *M. b*-NiCu@CdS for CO_2_-to-CH_4_ conversion is evaluated as the ratio of the product free-energy change to the input solar energy. The calculated value is ~0.7%, comparable to the energy efficiency of photosynthesis occurring in most plants^32^. Moreover, the overall energy efficiency of *M. b*-NiCu@CdS could be higher when considering the Gibbs free energy gains (Δ_*r*_*G*^*o*^) from CO_2_ to biomass due to the significant increase of cell concentration with a high live/dead ratio (Supplementary Fig. 4). The excellent CH_4_ yield obtained with *M. b*-NiCu@CdS remains stable over three 5-day cycles (i.e., 15 days in total, Supplementary Fig. 16), suggesting the potential for practical application.

### Function of interfacial binary active sites

**Fig. 4:**
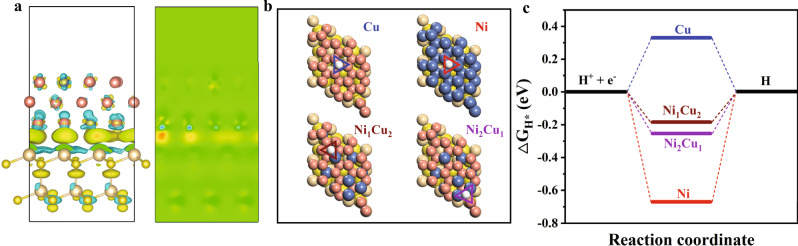
DFT calculation. **a** Differential charge density at the interface between NiCu and CdS, and the corresponding planar averaged charge density difference. Yellow and blue colours represent an increase and decrease in electron density, respectively. **b** Possible active sites for the independent adsorption of H atoms. **c** Free-energy diagrams for hydrogen evolution pathways. Dark blue, pink, light brown, yellow and white balls represent Ni, Cu, Cd, S and H atoms, respectively. Source data are provided as a Source Data file.

The electron transfer and capture at the biotic-abiotic interface are a key process in the methanogen-semiconductor hybrid system. Due to the fast recombination of electron-hole pairs in CdS and slow extracellular electron uptake by *M. b* metabolism^33^, significant energy losses and side reactions occur in the *M. b*-CdS-based hybrid systems, resulting in a poor CH_4_ selectivity^12^. To elucidate the role of binary NiCu sites on the electron transfer at the biotic-abiotic interface, density functional theory (DFT) calculations are conducted to investigate the interfacial behaviour between CdS and NiCu alloy, which then influence the electron capture and methanogenesis of *M. b*. Considering the alloy nature of NiCu, an optimal DFT model for the alloy structure is developed (Supplementary Fig. 17). The difference in work functions of CdS and NiCu alloy results in the Mott-Schottky effect at the interface. As a result, the photoexcited electrons in the conduction band of CdS can transfer to the NiCu alloy, followed by electron redistribution. This feature is further confirmed by the differential charge densities in the presence of NiCu alloy (Fig. 4a). The surface charges of CdS (111) are depleted, while the charges are accumulated at the interface, suggesting the charge transfer from CdS to the NiCu alloy. The improved transfer of photoexcited electrons with NiCu@CdS results in an enhanced photocurrent intensity, reduced electrochemical impedance, and suppressed photoluminescence (PL) along with a prolonged decay time (Supplementary Fig. 18 and Supplementary Table 6).

Upon recognizing the role of NiCu in facilitating electron transfer, we further examine the hydrogen transfer in the system. The binding energies of hydrogen atoms at various adsorption sites are calculated (Fig. 4b). As shown in Fig. 4c, the binding energy of hydrogen atom at the hollow site of Ni_1_Cu_2_@CdS (Ni-Cu-Cu@CdS) is close to the optimum value (i.e., 0 eV) and the benchmark value of Pt (i.e., –0.09 eV). It turns out that the replacement of one surface Cu with a Ni atom results in an optimal surface, while a lower or higher Ni concentration would lead to an insufficient number of active sites or the formation of inactive sites. Once H atoms are formed from water or protons by photoexcited electrons, the optimal H adsorption strength favours the transfer of H atoms from Ni-Cu-Cu sites to elsewhere (i.e., *M. b* in our case). Taken together, with the integration of electron and hydrogen transfer abilities, the interfacial NiCu alloy can promote the methanogenesis of *M. b* while avoiding H_2_ production as a side reaction.

### Mechanism for methanogenesis in biotic-abiotic system

**Fig. 5:**
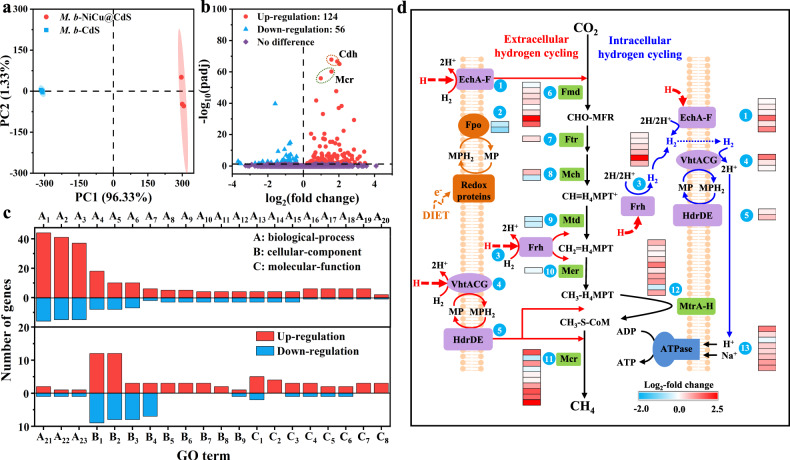
Transcriptomic analyses. **a**, **b** PCA (**a**) and differential gene volcano graph (**b**) of *M. b*-NiCu@CdS as compared with *M. b*-CdS. **c** The top 40 genes with significant enrichment analysed by GO enrichment of differentially expressed genes. A, B and C represent biological processes, molecular functions and cellular components, respectively. A1 biosynthetic process, A2 organic substance biosynthetic process, A3 cellular biosynthetic process, A4 organonitrogen compound biosynthetic, A5 ion transport, A6 cation transport, A7 ion transmembrane transport, A8 cellular respiration, A9 energy derivation by oxidation of organic compounds, A10 anaerobic respiration, A11 methane metabolic process, A12 methanogenesis, A13 energy derivation by oxidation of reduced inorganic compounds, A14 cellular alkane metabolic process, A15 alkane biosynthetic process, A16 proton transmembrane transport, A17 inorganic ion transmembrane transport, A18 inorganic cation transmembrane transport, A19 cation transmembrane transport, A20 porphyrin-containing compound biosynthetic process, A21 porphyrin-containing compound metabolic process, A22 methanogenesis, from acetate, A23 regulation of response to stress; B1 cation binding, B2 metal ion binding, B3 vitamin binding, B4 cation transmembrane transporter activity, B5 coenzyme-B sulfoethylthiotransferase activity, B6 transferase activity, transferring alkylthio groups, B7 transferase activity, transferring sulfur-containing groups, B8 serine-type endopeptidase inhibitor activity, B9 2-isopropylmalate synthase activity; C1 organelle membrane, C2 proton-transporting two-sector ATPase complex, C3 mitochondrial membrane, C4 mitochondrial envelope, C5 mitochondrial inner membrane, C6 organelle inner membrane, C7 proton-transporting V-type ATPase complex, C8 proton-transporting two-sector ATPase complex, proton-transporting domain. **d** Differential expression analysis of key genes for methanogenesis with *M. b*-NiCu@CdS compared with *M. b*-CdS. (1) EchA-F hydrogenases, (2) F_420_H_2_ dehydrogenases (Fpo), (3) F_420_-dependent hydrogenases (Frh), (4) MP-reducing hydrogenases (VhtACG), (5) Heterodisulfide reductases (HdrDE), (6) Formylmethanofuran dehydrogenases (Fmd), (7) Formylmethanofuran-tetrahydromethanopterin N-formyltransferase (Ftr), (8) Methenyltetrahydromethanopterin cyclohydrolase (Mch), (9) Methylenetetrahydromethanopterin dehydrogenase (Mtd), (10) 5,10-methylenetetrahydromethanopterin reductase (Mer), (11) Methyl-CoM reductases (Mcr), (12) Methyltransferases (MtrA-H), (13) ATP synthase. Source data are provided as a Source Data file.

To gain insight into the methanogenesis in our system, we investigate the response of the complex energy-transferring networks of *M. b* to the hydrogen transfer through NiCu@CdS, using transcriptomic analyses. Principal component analysis (PCA) shows two distinct groups in the score plots for *M. b*-CdS and *M. b*-NiCu@CdS, indicating that the NiCu alloy remarkably influences the physiological state of *M. b* under light irradiation (Fig. 5a). A total of 124 genes are significantly upregulated (fold change > 1.20 and *p* < 0.05), while 56 genes are significantly downregulated (fold change > 1.20 and *p* < 0.05) in response to the introcorporation of NiCu alloy (Fig. 5b). These genes encode various processes and characteristics, such as biological processes, molecular functions and cellular components (Fig. 5c). In particular, the Hsp70 (DnaK-DnaJ-GrpE chaperone system) and Hsp60 (GroEL) systems have been reported to serve as indicators of environmental stress in *M. b*^34^. Surprisingly, the related genes of the Hsp70 and Hsp60 systems are not significantly regulated in our case, implying that our biotic-abiotic hybrid system maintains a suitable environment for the cell growth and metabolism of *M. b* after the involvement of NiCu. This feature could be attributed to the significantly increased excretion of extracellular polymeric substances (EPS) with the higher fluorescence intensities after the introduction of NiCu (Supplementary Fig. 19). Specifically, these EPS components, including proteins, polysaccharides and lipids, can bind with cells through complex interactions to form a vast net-like structure that protects cells against the harm of external tress^35,36^. Meanwhile, the increased EPS concentration, particularly the proteins as the most prominent EPS component, could effectively bind with metal cations (i.e. Cu^2+^ and Ni^2+^) to act as a permeability barrier to hinder the intracellular penetration of excessive metal ions and potential DNA damage^37^. This argument is further confirmed by a higher concentration of membrane-bound protein in the *M. b*-NiCu@CdS hybrid system (Supplementary Fig. 20). In addition, many components of EPS are redox-active or electrically conductive^38^, and as such, their combination with NiCu is beneficial for the extracellular electron transfer from NiCu into the cell for the growth and metabolism of *M. b* in the *M. b*-NiCu@CdS hybrid system. This finding is further verified by the insignificant change in the transcription level of DNA-binding protein from starved cells (Dps), which is known to protect cells against various types of stressors such as starvation, oxidative stress, metal toxicity, thermal stress and salt stress^39^. In addition, the nickel transport proteins (CbiN and CbiM, *p* < 0.05) in *M. b*-NiCu@CdS are significantly upregulated, which could be evidence of an effective interaction between the surface of *M. b* and Ni atoms^40^. In fact, the living cells still maintain highly similar overall morphology to that of bare *M. b* after integrated NiCu@CdS, and remain close contact with the NiCu@CdS after light irradiation (Supplementary Fig. 21), demonstrating the stability of the self-assembled structure.

Furthermore, the transcriptional levels of the energy-converting ferredoxin-dependent hydrogenase (Ech), F_420_-reducing hydrogenase (Frh) and membrane-bound methanophenazine-dependent hydrogenase (Vht) are significantly upregulated with *M. b*-NiCu@CdS (Fig. 5d). This contributes to enhanced hydrogenotrophic methanogenesis, with the transferred H atoms (and possibly a small amount of produced H_2_ below the threshold concentration) as electron donors, through the extracellular hydrogen cycle. These hydrogenases are also involved in the intracellular hydrogen cycle, in which the cooperative activity of Vht and HdrDE generates a proton gradient across the cell membrane. As such, the released energy in this gradient is captured by ATP synthase, resulting in the increased transcriptional levels. These results provide a further explanation for the superior methanogenesis performance of *M. b*-NiCu@CdS where all methanogenesis genes show upregulated expression patterns, particularly the unique and ubiquitous *Mcr* genes in methanogens encoding the terminal step of CH_4_ generation (Fig. 5d). Moreover, it should be noted that no significant change is detected in the expression of different subunits of the membrane-bound F_420_ dehydrogenase (Fpo), which is generally considered to receive electrons via the DIET pathway for the generation of reduced F_420_. This indicates that hydrogen atom is the preferred and main intermediate for methanogenesis with *M. b*-NiCu@CdS.

The ultrafast transient absorption (TA) spectroscopy is further employed to examine the photoexcited electrons transfer in the *M. b*-NiCu@CdS hybrid system. Similarly to the typical spectra of CdS-related systems^15^, a visible band peaking at 430–500 nm is observed for the NiCu@CdS-related systems after excitation, which is absent in the NiCu@CdS-free *M. b* system (Supplementary Fig. 22). Fitting of the TA data sets to a biexponential decay reveals that the transfer of photoexcited electrons in the *M. b*-NiCu@CdS-related hybrid systems is governed by the multiple processes (Supplementary Table 7). Compared with the NiCu@CdS system, an accelerated decay kinetics of photoexcited electrons is observed after the introduction of *M. b*, possibly attributed to the more e^-^ acceptor sites available on the surface of *M. b*, such as the high activity of hydrogenase (H_2_ase). To further confirm our assumption, the activity of H_2_ase is inhibited by CO to evaluate its role on the transfer and capture of photoexcited electrons. Differently from the previous results^15,41^, the increasing TA lifetimes with CO suggest that NiFe active sites in H_2_ase are the important electron acceptor sites. In addition, further inhibition experiment shows that the electron decay kinetics is dramatically decelerated by the addition of diphenyleneiodonium (DPI) to inhibit the CoB-S-S-CoM-dependent oxidation of reduced cytochromes and H_2_-dependent cytochrome reduction^42^. Taken together, these evidences demonstrate that the membrane-bound H_2_ase and cytochromes are responsible for the transfer of photoexcited electrons in *M. b*-NiCu@CdS hybrid systems.

Based on the information gleaned above, the pathways for electron and proton transfer pathway toward CH_4_ production with the *M. b*-NiCu@CdS are proposed (Supplementary Fig. 23). Due to the intimate contact, a Fermi level equilibrium (–0.76 V) can be achieved in the NiCu@CdS. Under light irradiation, electrons are first photoexcited from the valance band (VB, 1.68 V) to the conduction band (CB, –1.00 V) of CdS, and then transferred to NiCu alloy (–0.76 V) across the interface. As the reduction potential of the NiCu alloy is higher than those of the H_2_ase and cytochrome *b*^43^, the efficient electron transfer is subsequently conducted from the NiCu alloy to the acceptor sites on the surface of *M. b* for methanogenesis. This electron transfer channel can effectively hinder the backflow of photoexcited electrons to recombine with holes, and then improve the efficiencies of charge separation and utilization. Meanwhile, the hydrogen at the NiCu@CdS interface, produced via the interaction between the electrons and protons from the oxidation of H_2_O and cysteine, can be harvested by *M. b* to significantly improve the CH_4_ production through the extracellular and intracellular hydrogen cycling.

## Discussion

A quantum yield of 85 ± 12% was observed with the *M. thermoacetica*-CdS hybrids for the photosynthesis of acetic acid from CO_2_ in a previous work^10^. However, it should be pointed out that the value was achieved under the very low photon flux (the order of magnitude of ×10^13 ^cm^−2^ s^−1^) that tried to reduce losses from charge recombination, along with the high hybrid loading (four times). When the photon flux increased to 1.6 × 10^15 ^cm^−2^ s^−1^, the quantum yield dramatically dropped to 4 ± 1%. In particular, the peak quantum yield further decreased to only 2.44 ± 0.62% under low-intensity simulated sunlight with a light-dark cycle of 12 hour each, which was order-of-magnitude lower than that in our work (12.41 ± 0.16%, 1.6 × 10^15^ cm^−2^ s^−1^). Therefore, this study addresses the long-standing challenge via the bimetallic alloy strategy to achieve a high quantum yield under relatively high photon flux. More importantly, these results demonstrate the potential of *M. b*-NiCu@CdS hybrid system for indoor application that has the similar light intensity (<1 mW cm^−2^)^44^ to that reported in this study (0.8 mW cm^−2^). To this end, energy harvesting for hybrid system from the indoor light sources, such as light emitting diodes (LEDs), fluorescent lamps or halogen lamps, not only brings opportunities to directly supply the high-grade biogas for daily life without the complex transmission pipeline system, but also meets the demand of zero net energy (ZNE) building in the future. To further evaluate the practicability of the hybrid system, we illuminate *M. b*-NiCu@CdS under simulated sunlight (AM 1.5 G, 20 mW cm^−2^) with a light-dark cycle of 12 h. A quantum yield of 1.01% of total incident simulated sunlight is observed after 3-day operation, which is order-of-magnitude comparable to the year-long averages determined for plants and algae (~0.2 to 1.6%)^10^. With the further increasing light intensity, the methanogenesis performance dramatically decreases. The quantum yield is only 0.05% under 100 mW cm^−2^ after 3-day operation, most likely due to the photodamage of cells such as destruction of the cell membrane under a higher light intensity^11^. However, such a limitation does not affect the application of biohybrid system outdoors, because the shading technology, such as the use of optical glasses, shading nets and green house, becomes more and more mature.

While cysteine has been selected as a representative sacrificial hole scavenger (i.e., electron donor) in our study, we can potentially use other electron donors, including the low-value organic compounds such as lactic acid, formic acid and methanol^45–47^, and the universal inorganic electron donors such as sodium sulfide (Na_2_S) and sodium sulfide/sodium sulfite (Na_2_S/Na_2_SO_3_) to enable similar scenarios^48,49^. For instance, an excellent methanogenesis performance is achieved with the *M. b*-NiCu@CdS hybrid system after the replacement of cysteine with KI (+0.35 V vs. SHE at pH 7, Supplementary Fig. 14). Furthermore, the abundant wastes such as the lignocellulose and plastics may also be used as the potential electron donors^50,51^. As such, there is a broad electron donor spectrum to support the *M. b*-NiCu@CdS hybrid system, offering the possibility of realizing both high quantum efficiency and energy conversion efficiency that may play an important role in scalable energy-storage systems of the future^52^. In this sense, a different autotrophic way has been established for *M. b* methanogens via direct hydrogen transfer through interface engineering, differently from the traditional chemoheterotroph using organic carbon sources and chemoautotroph using H_2_. In addition, compared with *Moorella thermoacetica*-CdS, an increased acetate yield is also observed in the *Moorella thermoacetica*-NiCu@CdS hybrid system (Supplementary Fig. 24). For this reason, it can be predicted that such a promising alloy strategy is able to improve the CO_2_ conversion to various value-added products, not limited to CH_4_, which would open an avenue for achieving effective and scalable conversion of CO_2_ to biofuels.

## Methods

### Synthesis of nanoparticles

Ni_*x*_Cu_*y*_ alloys (where *x* and *y* refer to the molar ratio of Ni and Cu) were prepared through a galvanic replacement reaction. For instance, 0.8 mmol NiSO_4_·6H_2_O was dissolved in 150 mL of ultrapure water. Then Ni^2+^ was completely reduced to Ni^0^ after dropwise addition of potassium borohydride (KBH_4_, 0.4 M) until the bubbles and green colour disappeared under a mechanical stirring speed of 200 rpm at 25 °C. Afterward, 0.64 mmol CuSO_4_·5H_2_O was added dropwise into the mixture to partially replace Ni^0^. After 20 min of reaction, the typical blue colour of Cu^2+^ disappeared. Subsequently, the mixture was centrifuged at 5000 × *g* for 10 min, washed six times with ultrapure water, and freeze-dried for 48 h to obtain Ni_2_Cu_8_ alloy. To investigate the effect of hydrogen on methanogenesis performance, the produced Ni_2_Cu_8_ alloy was immersed in 100 mM 1,4-diphenylbutadiyne (DPB) solution for 12 h to form a layer of hydrogen scavenger before use^53^. The CdS semiconductor was synthesized according to the following protocols^54^, where 3-mercaptopropionic acid (MPA) was used to control the particle size of CdS nanoparticles. Briefly, 10 mmol of CdCl_2_ and 17 mmol of MPA were dispersed in 200 mL of water. The solution pH was adjusted to 10.0 by 5.0 mol L^−1^ of NaOH, and transferred into a 250 mL three-necked flask. Then the air in the headspace of the flask was replaced with Ar, and 0.5 mmol of freshly prepared Na_2_S solution was dropwise added under stirring. Subsequently, the mixture was heated to 100 ^o^C with a condenser, and the observed bright-yellow, transparent solution was continually stirred at 100 ^o^C for 30 min. After naturally cooled to room temperature, the CdS semiconductors were isolated through precipitation with alcohol, redispersed in water, and freeze dried for further use.

### Construction of biotic-abiotic hybrid system

*M. b* (DSM 800) was obtained from DSMZ (Braunschweig, Germany) and inoculated into 50 mL of heterotrophic medium at 37 °C with a ratio of 1:5 (Table S8). When the growth of *M. b* reached the logarithmic phase (OD_600_~0.2), 2.16 mg Ni_2_Cu_8_ alloy and 4.32 mg CdS semiconductor were added in turn over a time interval of 12 h according to the preliminary experiments. After a total of 24 h of cultivation at a mechanical stirring speed of 180 rpm, the suspension of *M. b*-(50%Ni_2_Cu_8_)@CdS (denoted as *M. b*-NiCu@CdS hereafter) was sequentially centrifuged, washed and resuspended in 5 mL of 0.9% NaCl solution three times for further use. As controls, *M. b*-Cu@CdS, *M. b*-Ni@CdS and *M. b*-CdS were prepared by the same method, except that NiCu@CdS was replaced with Ni@CdS, Cu@CdS and CdS, respectively. In addition, *Moorella thermoacetica* (*M. t*, ATCC39073) was obtained from the Global Bioresource Center ATCC, and inoculated into 50 mL of heterotrophic medium at 52 °C with a ratio of 1:5 (Table S9). Then *M. t-*CdS *and M. t-*NiCu@CdS were prepared by the same method, except that *M. b* was replaced with *M. t*.

### Characterization

The hybrid materials were fixed with 2.5% glutaraldehyde for 12 h, and dehydrated in 25, 50, 75, 90 and 100% ethanol sequentially^55^, the morphologies of the hybrid materials were observed with SEM (Hitachi SU8020) and high-resolution TEM (HRTEM, FEI Tecnai G2 F20 S-TWIN). Surface elemental compositions and states were analysed with EDS (X-MaxN, Oxford Instruments). XPS measurements were performed by a Thermo ESCALA 250 XPS spectrometer system using Al Kα (1486.6 eV) radiation (operated at 15 kV) in the constant analyzer energy mode. Fluorescence images were collected using confocal laser scanning microscopy (CLSM, Carl Zeiss LSM880). XRD patterns were obtained using an X-ray diffractometer (XRD-6000, Shimadzu) with Cu Kα radiation at 40 kV and 30 mA in a 2*θ* range of 5–80° at a scan speed range of 1° min^−1^. X-ray absorption spectroscopy (XAS) measurements for the Ni and Cu *K*-edges were conducted in transmission mode (fluorescence mode) on beamline 12-BM at the Advanced Photon Source at Argonne National Laboratory. The XANES and Fourier transformed EXAFS spectra were analysed using Athena and Artemis software. The zeta potentials of the samples were measured using a Nanobrook Omni analyser (Nano-brook Omni, Brookhaven, US).

Electrochemical characterization was conducted in a three-electrode anaerobic electrochemical quartz chamber connected to a CHI 660E electrochemical station (CH Instruments, TX, USA). The working electrode used was an indium tin oxide (ITO)-coated glass plate (radius: 1.5 cm) placed at the bottom of the chamber with 6.48 mg of self-precipitated samples. A platinum sheet (1 × 1 cm) and a saturated calomel electrode (SCE) mounted on the salt bridge were used as the counter and reference electrodes, respectively. The photocurrents (*I-t*) were measured under a light on/off cycle of 60 s at a bias potential of –300 mV versus SCE. Electrochemical impedance spectroscopy (EIS) experiments were conducted from 1000 kHz to 0.01 Hz with a sinusoidal perturbation amplitude of 5 mV under an open-circuit potential. Steady-state PL spectra and PL decay spectra were determined by a PL spectrometer (FLS980, Edinburgh Instruments), and the lifetime data were analysed with DataStation V6.6 (Horiba Scientific).

### Photocatalytic experiments

Methanogenesis experiments were conducted in 125 mL anaerobic serum bottles that were sealed with Teflon®-coated rubber and aluminium crimp caps. 5 mL of different hybrid materials was added to 50 mL of sterilized autotrophic medium (Supplementary Table 8) with CO_2_ as the sole sources by bubbling the headspace with the filter-sterilized N_2_/CO_2_ (80:20) gas mixture for 10 min at a rate of 5 mL min^−1^. Meanwhile, cysteine was added into each bottle to act as a sacrificial reducing agent. Then the bottles were maintained at 37 °C in a constant temperature incubator (LRH-1500F, Shanghai Bluepard Instrument Co., China) for light-driven methanogenesis experiments. The Ni/NiCu and NiCu/CdS ratios, NiCu@CdS dosage and Cys concentration in the biotic-abiotic hybrid system with *M. b*-NiCu@CdS were sequentially optimized. As a control, the *M. b* in the hybrid system was inactivated, and the related H_2_ evolution efficiency and CH_4_ yield were evaluated. Moreover, isotopic labelling experiments were conducted using NaH^13^CO_3_ and D_2_O instead of NaH^12^CO_3_ and H_2_O in the medium, respectively, and the headspace of anaerobic serum bottles were bubbled with the filter-sterilized N_2_ gas for 10 min at a rate of 5 mL min^−1^. To mimic natural day-night cycles, the methanogenesis performance with *M. b*-NiCu@CdS was investigated with 12-hour light-dark cycles. The medium was refreshed to evaluate the stability of *M. b*-NiCu@CdS. Furthermore, the methanogenesis performance with KI as sacrificial reagent was evaluated. The experiments for CO_2_-to-acetate conversion were conducted with the same protocols in 50 mL of sterilized autotrophic medium (Supplementary Table 9) at 52 ^o^C. The light source for all photocatalytic experiments was 395 ± 5 nm LEDs (0.8 ± 0.1 mW cm^−2^) or a collimated 75 W Xenon lamp (CEL-HXF300, Ceaulight, China) with an AM 1.5 G filter.

### Product measurements

The H_2_ and CH_4_ concentrations were determined using an Agilent 7890 A Gas Chromatograph (GC) equipped with a flame ionization detector (FID). The gas products in the headspace of the hybrid system during the isotopic labelling experiments were determined by a Shimadzu GC-2010 equipped with a Shimadzu AOC-20i auto sampler system and interfaced with a Shimadzu QP 2010S mass spectrometer (DB-5 capillary column (30 m×0.25 mm × 0.25 μm), Helium (99.999%) as carrier gas with a flow rate of 1.2 mL min^−1^). Acetate measurement was performed on a Shimadzu Nexis GC-2030 system equipped with a CP Wax 52 CB column and an FID detector. The metabolites of cysteine by *M. b* were characterized by Varian INOVA 600-MHz NMR spectroscopy (Varian, USA).

Both *M. b*-NiCu@CdS and *M. b*-CdS samples after light irradiation for 72 h were collected and centrifuged at 10,000 × *g* for 30 min at 4 °C for transcriptomic analysis^56^. Total RNA was extracted with TRIzol reagent (Invitrogen, California, USA). Directional multiple libraries were prepared with the NEBNext® Ultra II™ Directional RNA Library Prep Kit (New England Biolabs Ltd., Beijing, China). rRNA was depleted using subtractive hybridization employing biotinylated rRNA probes. All reads matching 16S and 23S rRNA genes were removed, and the remaining reads were mapped against the published genome of *M. b* MS (NZ_CP009528.1). Mapped reads were normalized with FPKM (fragments per kilobase per million mapped reads) method. Multiple fluorescent staining was conducted with fluorescein-isothiocyanate (FITC) (proteins), concanavalin A (Con A, α-polysaccharide), calcofluor white (β-polysaccharide) and Nile red (lipids)^28^, and then observed with CLSM (Carl Zeiss LSM880). For the isolation of the extracellular membrane-bound proteins, the cell culture of *M. b* was subjected to shearing forces in a Waring blender at room temperature at a low speed for 1 min and measured with the BCA protein assay kit (Thermo Scientific Pierce). Cell viability were measured using the Live/Dead® BacLight^TM^ Bacterial Viability Kit, observed with CLSM (Carl Zeiss LSM880), and then analysed with ImageJ software^57^. The average quantum yield (*QY*) was defined by the ratio of the effective electrons used for CH_4_ production to the total input photon flux as the following equation^10,58^:

1 $$\begin{array}{c}QY(\%)=\frac{{{{{{\rm{the}}}}}}\,{{{{{\rm{number}}}}}}\,{{{{{\rm{of}}}}}}\,{{{{{\rm{electrons}}}}}}\,{{{{{\rm{accepted}}}}}}\,{{{{{\rm{for}}}}}}\,{{{{{{\rm{CH}}}}}}}_{4}\,{{{{{\rm{production}}}}}}}{{{{{{\rm{the}}}}}}\,{{{{{\rm{number}}}}}}\,{{{{{\rm{of}}}}}}\,{{{{{\rm{incident}}}}}}\,{{{{{\rm{photons}}}}}}}\times 100\%\\=\frac{[8\times C({{{{{{\rm{CH}}}}}}}_{4})]\times V\times 6.02\times {10}^{23}}{\frac{{P}_{{{{{{\rm{ligh}}}}}}t}At\lambda }{hc}}\times 100\%\end{array}$$

where *C*(CH_4_) is the CH_4_ concentration (mol L^−1^), *V* is the volume of the reactor headspace (L), *A* is the area of irradiation (15.9 cm^2^), *t* is the illumination time, *λ* is the light wavelength (395 nm), *h* is the Planck constant (6.63 × 10^−34 ^J s^−1^), *c* is the speed of light (3 × 10^17 ^nm s^−1^), and *P*_light_ is the light intensity (0.8 mW cm^−2^).

The energetic efficiency ($\eta$) was calculated as the ratio of the product free-energy change to the input solar energy by the following equation^52^:

2 $$\eta=\frac{{{\mbox{Energy}}}\,{{\mbox{stored}}}}{{{\mbox{Energy}}}\,{{\mbox{Input}}}}=\frac{N*{\triangle }_{r}{G}^{o}}{{E}_{{{{{{\rm{solar}}}}}}}}$$

where Δ_*r*_*G*^*o*^ is the reaction Gibbs free-energy change, *N* is the number of moles of produced CH_4_, and *E*_solar_ is the total incident photon energy from the simulated sunlight.

With the stoichiometry of photooxidation of I^-^ into I_3_^-^ (3I^−^ + 2 h^+^ → I_3_^−^), a maximum CH_4_ yield of 0.075 mmol could be achieved, corresponding to 0.9 mmol KI. CH_4_ efficiency was then defined as:

3 $${{{{{\rm{Efficiency}}}}}}\,(\%)=\frac{{C}_{{{{{{\rm{a}}}}}}}}{0.075}\times 100\%$$

where *C*_a_ is the CH_4_ yield (mmol).

The ultrafast femtosecond transient absorption (fs-TA) measurements were conducted on a Helios spectrometer system (Ultrafast systems, USA) with a modelocked Ti:sapphire seed laser (Spectra Physics, Maitai) directed to a regenerative amplifier (Spitfire Pro, Spectra Physics) and a high power laser (Empower, Spectra Physics). Briefly, the samples were excited with ∼120-fs laser pulses at a repetition rate of 1 kHz. A chopper that can modulate the pump pulses was employed to obtain fs-TA spectra with and without the pump pulses alternately, and an optics fibre coupled to a multichannel spectrometer with a CMOS sensor was used to record the pump-induced fluctuation in probe/reference beam intensity with adjusting the optical delay line (maximum ~3 ns). The obtained spectra were further processed by the Surface Xplorer, and fitted to a multiexponential decay.

All culture and sampling manipulations were performed using the sterile technique in a Vacuum Atmospheres Nexus One glovebox with a N_2_ atmosphere (H_2_O < 1 ppm, O_2_ < 1 ppm). All experiments were performed at least in triplicate. Student’s *t* test was used to evaluate the differences, and a *p* value < 0.05 was considered statistically significant.

### Computational details

All the calculations were performed in the framework of the density functional theory with the projector augmented plane-wave method, as implemented in the Vienna ab initio simulation package^59^. The generalized gradient approximation proposed by Perdew, Burke and Ernzerhof was selected for the exchange-correlation potential^60^. The cut-off energy for plane wave was set to 450 eV. The energy criterion was set to 10^−5^ eV in iterative solution of the Kohn-Sham equation. A vacuum layer of 15 Å was added perpendicular to the sheet to avoid artificial interaction between periodic images. The Brillouin zone integration was performed using a 3×3×1 k-mesh. All the structures were relaxed until the residual forces on the atoms had declined to less than 0.03 eV/Å.

The CO_2_ adsorption energy (Δ*E*_ads_) and free energy changes (Δ*G*) of reaction intermediates could be calculated by the following equations:

4 $$\varDelta {E}_{{{{{{\rm{ads}}}}}}}={E}_{{{{{{\rm{ads}}}}}}\ast }-{E}_{{{{{{\rm{ads}}}}}}}-{E}_{{{{{{\rm{slab}}}}}}}$$

5 $$\varDelta G=\varDelta E+\varDelta {E}_{{{{{\rm{ZPE}}}}}}-T\varDelta S$$

where *E*_ads*_ is the energy of CO_2_ adsorbed on the substrate, *E*_ads_ and *E*_slab_ are the energy of CO_2_ and substrate, respectively. The Δ*E* is the adsorption energy on the cluster surface from DFT calculations. The Δ*E*_ZPE_ and Δ*S* are the difference for the zero-point energy and entropy. The zero-point energy and entropy were calculated at the standard conditions corresponding to the pressure of 101,325 Pa (~1 bar) of H_2_ at the temperature of 298.15 K (*T*).

### Statistics and reproducibility

Each of the captured images presented in Fig. 2b–h, Supplementary Fig. 1b–f, Supplementary Fig. 4a, Supplementary Fig. 6b, Supplementary Fig. 19a and Supplementary Fig. 21 was measured independently for 5 times to confirm results reproducibility.

### Reporting summary

Further information on research design is available in the Nature Research Reporting Summary linked to this article.

## Supplementary information

Supplementary Information

Reporting Summary

## Data Availability

The authors declare that all data supporting the findings of this study are available in the article and its Supplementary Information. Source data are provided with this paper.

## Source data

Source Data
