# Supplementary material for: Solar-driven methanogenesis with ultrahigh selectivity by turning down H2 production at biotic-abiotic interface
Source: Nat Commun. 2022 Nov 3;13:6612. doi: 10.1038/s41467-022-34423-1 (PMC9633801; doi:10.1038/s41467-022-34423-1)
Supplement: Supplementary file 1 — Supplementary Information [file 41467_2022_34423_MOESM1_ESM.pdf]

Supplementary Information

Solar-driven methanogenesis with ultrahigh selectivity by turning down H_2 production at biotic-abiotic interface

Jie Ye[1], Chao Wang[1], Chao Gao[2], Tao Fu[1], Chaohui Yang[1], Guoping Ren[1], Jian Lü[1], Shungui Zhou[1]* & Yujie Xiong[2]*

[1]Fujian Provincial Key Laboratory of Soil Environmental Health and Regulation, College of Resources and Environment, Fujian Agriculture and Forestry University, Fuzhou 350002, China

[2]School of Chemistry and Materials Science, University of Science and Technology of China, Hefei 230026, China

*Corresponding authors.

Email: sgzhou@fafu.edu.cn; yjxiong@ustc.edu.cn

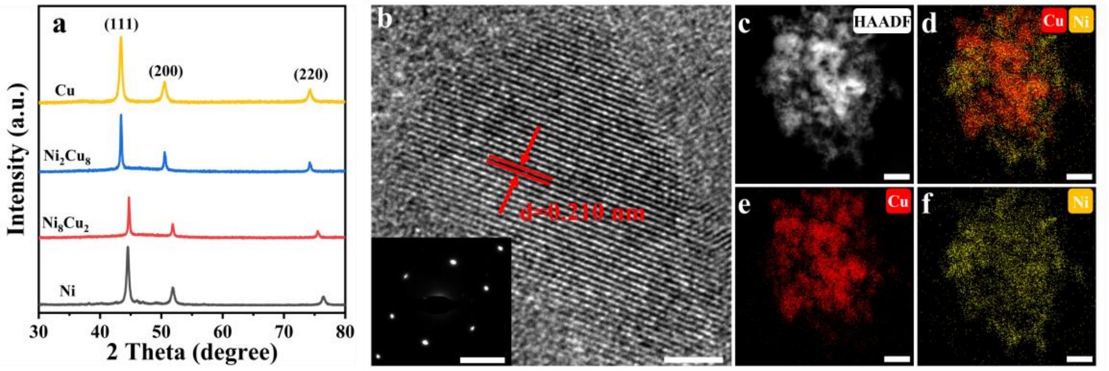

Supplementary Fig. 1 Structural characterization of NiCu alloys. (a) XRD patterns of NiCu alloys with different compositions. (b) HRTEM image (the inset image shows SAED pattern), and (c–f) EDX elemental mapping images of Ni_2Cu_8. Scale bars: 2 nm in b, 5 nm^{-1} in inset image, and 100 nm in c–f. Source data are provided as a Source Data file.

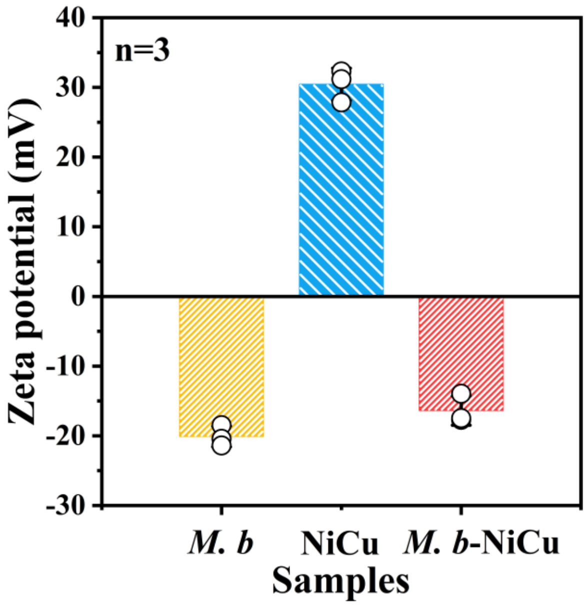

Supplementary Fig. 2 Zeta potentials of *M. b*, NiCu and *M. b*-NiCu. Data are presented as mean values ± standard deviation derived from n = 3 independent experiments. Source data are provided as a Source Data file.

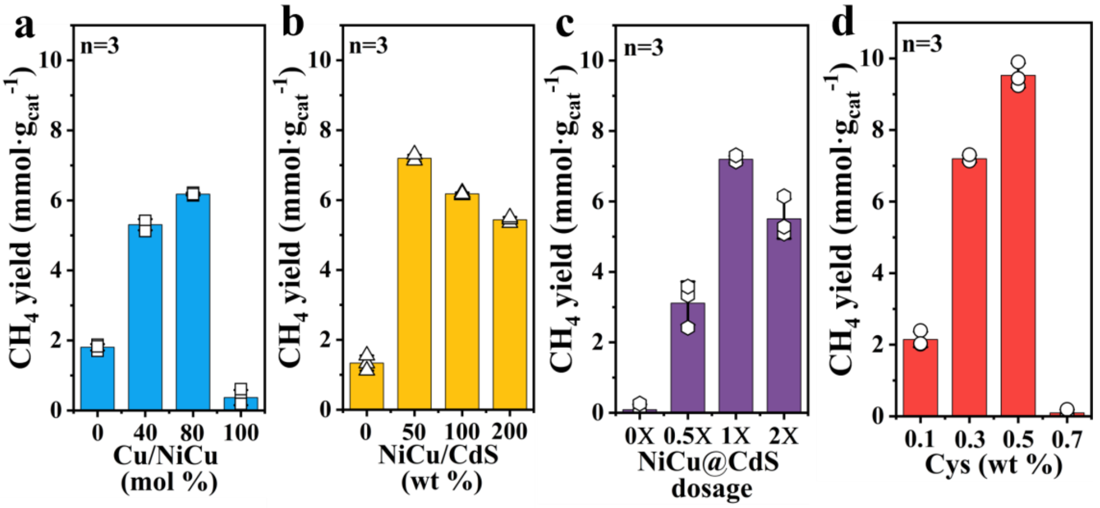

Supplementary Fig. 3 Methanogenesis performance of *M. b*-NiCu@CdS. Optimization of (a) Cu/NiCu ratio, (b) NiCu/CdS ratio, (c) NiCu@CdS loading and (d) cysteine (Cys) concentration for methanogenesis. Data are presented as mean values ± standard deviation derived from n = 3 independent experiments. Source data are provided as a Source Data file.

The methanogenesis performance of the well-designed *M. b*-NiCu@CdS biotic-abiotic hybrid system is optimized by considering the Cu/NiCu ratio, NiCu/CdS ratio, NiCu@CdS dosage and Cys concentration. Suitable Ni/Cu and NiCu/CdS ratios are beneficial for the balance between photoexcited electrons production/transfer and surface catalytic reactions. In particular, a volcano-like trend for CH_4 yield is observed with increase in NiCu@CdS dosage or cysteine concentration because the excessive Cd^{2+} from NiCu@CdS could inhibit the proliferation of *M. b*. In addition, too low cysteine concentration would result in its quick depletion, the subsequent serious photooxidative dissolution of CdS, and then the oxidative photodamage of cells[1]. However, too high cysteine concentration would lead to a significant decrease of pH that may inhibit the activity of methanogenic archaea[2]. A relatively stable pH value of 7.0 is adjusted during the preparation of *M. b*-NiCu@CdS hybrid system, and this value decreases after the addition of cysteine (Supplementary Fig. 4a). Although *M. b* was reported to be tolerant to the relatively low pH[3], the decrease of pH from 5.49 (0.5 wt% cysteine) to 3.53 (0.7 wt% cysteine) is seriously

inhibitory to its growth (Supplementary Fig. 4b). This is further confirmed by the growth experiments that no significant increase of OD_{600} value and CH_4 yield is observed with 0.7 wt% cysteine when acetate is used as the sole carbon source (Supplementary Fig. 4c and 4d). These results are consistent with the previous study that too low pH negatively influenced the microbial growth[4]. Furthermore, when more NiCu@CdS is used during the preparation of *M. b*-NiCu@CdS, the concentration of reactive oxygen species (ROS) produced under light irradiation is increased, posing more severe oxidative stress to *M. b*[5,6]. It turns out that a maximal CH_4 yield of 9.53 ± 0.33 mmol g_{cat}^{-1} is achieved with *M. b*-(50%Ni_2Cu_8)@CdS-0.5 wt% Cys.

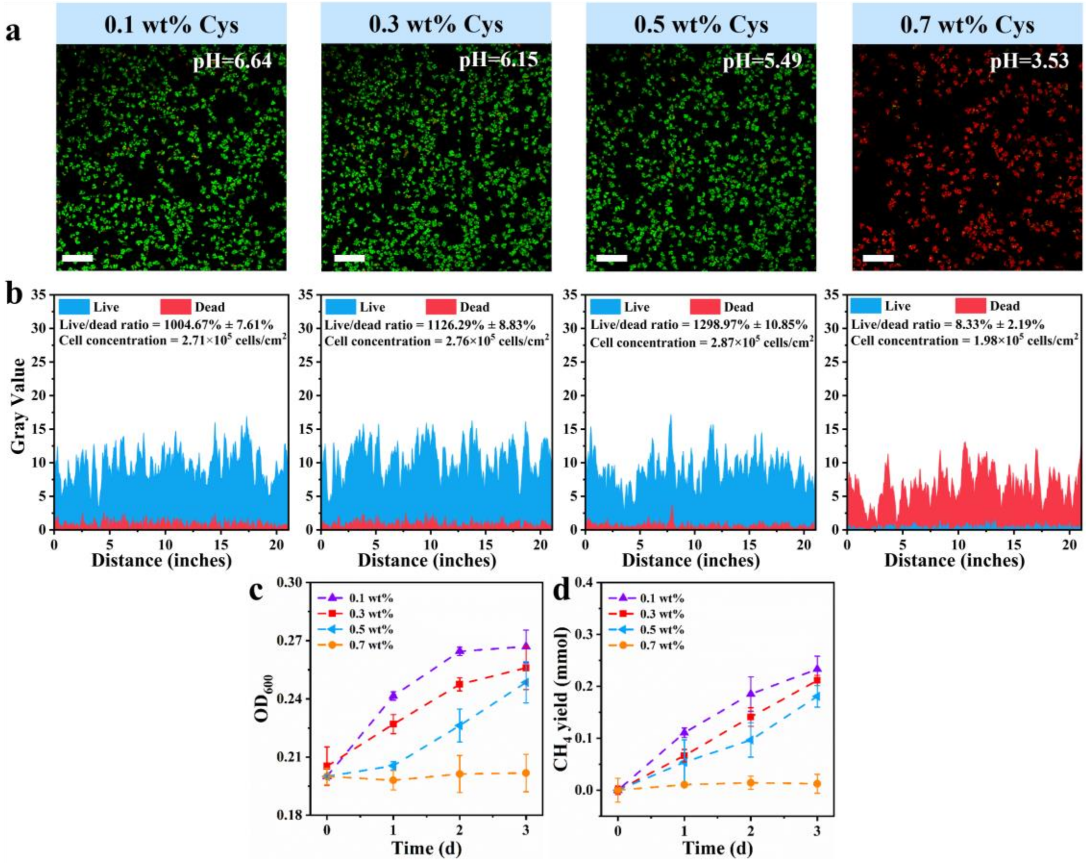

Supplementary Fig. 4 Viability of *M. b* with different concentration of cysteine. (a) CLSM images with the LIVE/DEAD BacLight viability staining in *M. b*-NiCu@CdS hybrid systems, and (b) the corresponding live/dead cell ratio and total cell concentration evaluated with Image J software. (c) Growth of *M. b* and (d) the corresponding CH_4 yields with acetate as the sole carbon source. *M. b* is grown in 50 mL sterilized heterotrophic medium with a ratio of 1:5 at 37 °C. Scale bars: 50 μm in a. Data are presented as mean values ± standard deviation derived from n = 3 independent experiments. Source data are provided as a Source Data file.

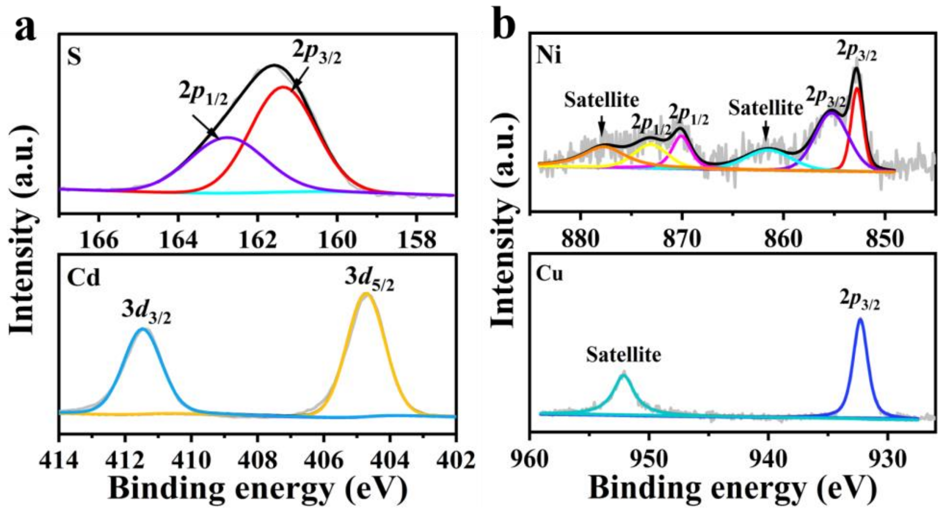

Supplementary Fig. 5 X-ray photoelectron spectra (XPS) for *M. b*-NiCu@CdS. (a) S 2p and Cd 3d and (b) Ni 2p and Cu 2p. Source data are provided as a Source Data file.

The compositions of *M. b*-NiCu@CdS are further corroborated by the XPS spectra, in which the peaks of Cd $3d_{5/2}$ at 404.6 eV and Cd $3d_{3/2}$ at 411.4 eV are associated with Cd^{2+} species[7]. The characteristic peaks of S $2p_{3/2}$ at 161.5 eV and S $2p_{1/2}$ at 162.8 eV are assigned to S^{2-} species[8,9]. In the Cu 2*p* spectra, a peak at 933.2 eV is observed, which can be attributed to metallic Cu[10]. The Ni 2*p* XPS spectra indicate that Ni^0 is also present in the system, which can be attributed to the highly dispersed metallic Ni[11].

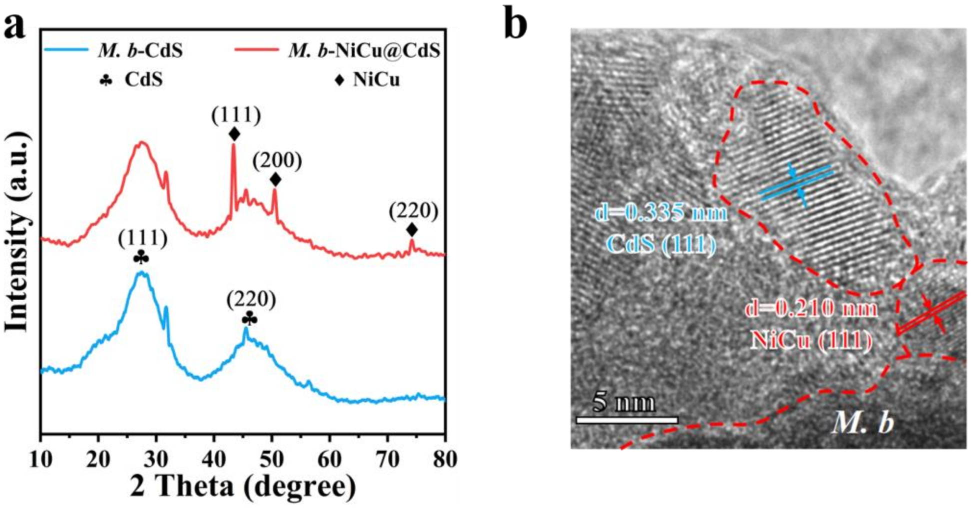

Supplementary Fig. 6 Structural characterization of *M. b*-CdS-based hybrid. (a) X-ray diffraction (XRD) patterns. (b) High-resolution transmission electron microscopy (HRTEM) image of *M. b*-NiCu@CdS. Source data are provided as a Source Data file.

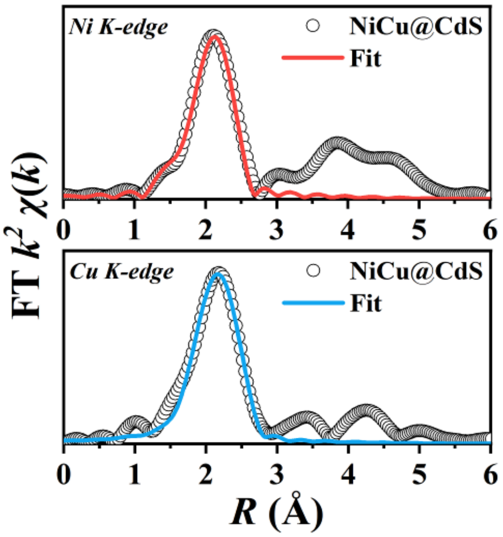

Supplementary Fig. 7 The corresponding extended X-ray absorption fine structure (EXAFS) *R*-space fitting curves. Source data are provided as a Source Data file.

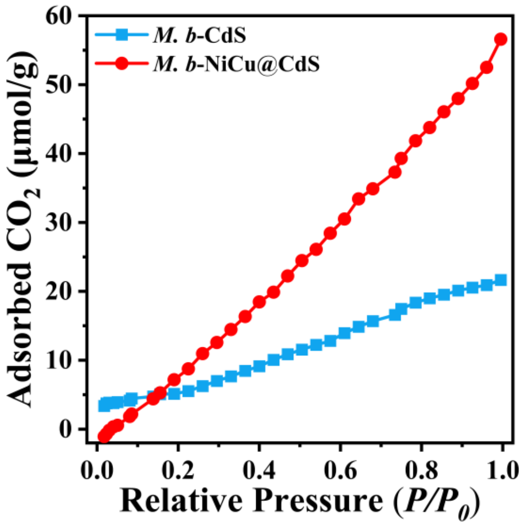

Supplementary Fig. 8 CO_2 adsorption isotherms of *M. b*-CdS and *M. b*-NiCu@CdS at 25 °C. Source data are provided as a Source Data file.

Typically, the CO_2 conversion process starts with the adsorption of CO_2 molecules on the catalyst surface. At 1 atm and 25 °C, the CO_2 adsorption of *M. b*-NiCu@CdS is 56.58 μmol g^{-1}, 2.61 times higher than that of *M. b*-CdS (21.67 μmol g^{-1}). Most likely, the addition of NiCu alloy increases the specific surface area and porosity of the catalyst, enhancing the ability for adsorbing CO_2.

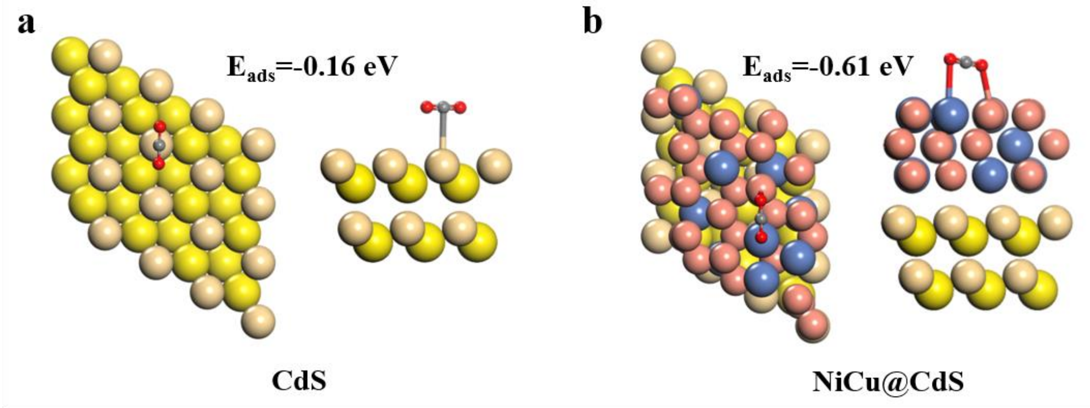

Supplementary Fig. 9 The favorable configurations and adsorption energies of CO_2 with (a) CdS and (b) NiCu@CdS.

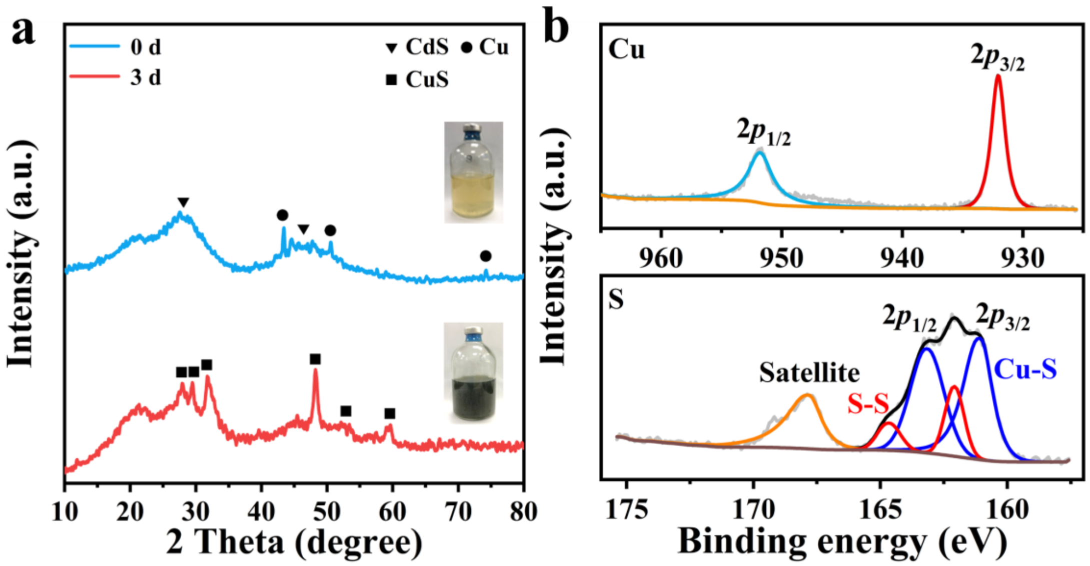

Supplementary Fig. 10 Structural characterization of Cu@CdS. (a) XRD patterns (inset: photographs of the hybrid system) and (b) XPS spectra of Cu@CdS after 3-day irradiation of 395 ± 5 nm LEDs (0.8 ± 0.1 mW cm^{-2}). Source data are provided as a Source Data file.

As shown in Supplementary Fig. 10a, the XRD pattern of the Cu@CdS after 3-day irradiation demonstrates the formation of CuS (JCPDS # 06-0464), in which the emerging peaks at 2θ = 27.90°, 29.46°, 31.74°, 48.24°, 52.72° and 59.50° are indexed to (101), (102), (103), (110), (108) and (116) planes, respectively[12,13]. The formation of CuS is further confirmed by the XPS spectra (Supplementary Fig. 10b) that the detected BE value of 951.79 and 932.05 eV for Cu 2p are associated with Cu^{2+} while the BE value of 161.06 and 163.15 eV for S 2p correspond to S^{2-} [12,14–16].

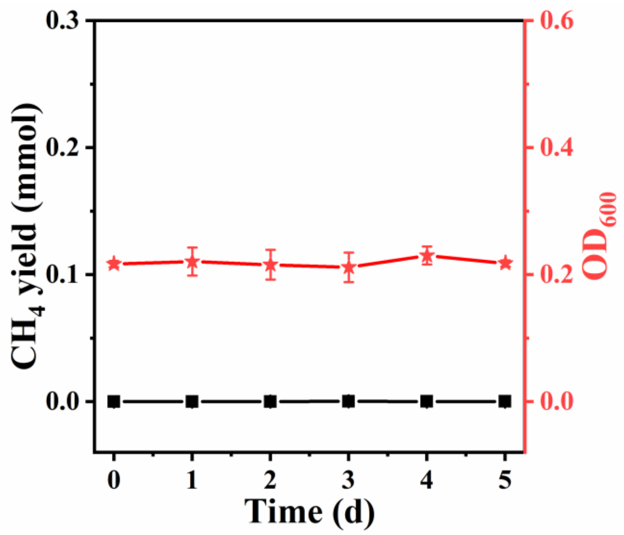

Supplementary Fig. 11 Growth of *M. b* and the corresponding CH_4 yield with cysteine as the potential carbon source. *M. b* is grown in 50 mL sterilized autotrophic medium with a ratio of 1:5 at 37 °C. Data are presented as mean values ± standard deviation derived from n = 3 independent experiments. Source data are provided as a Source Data file.

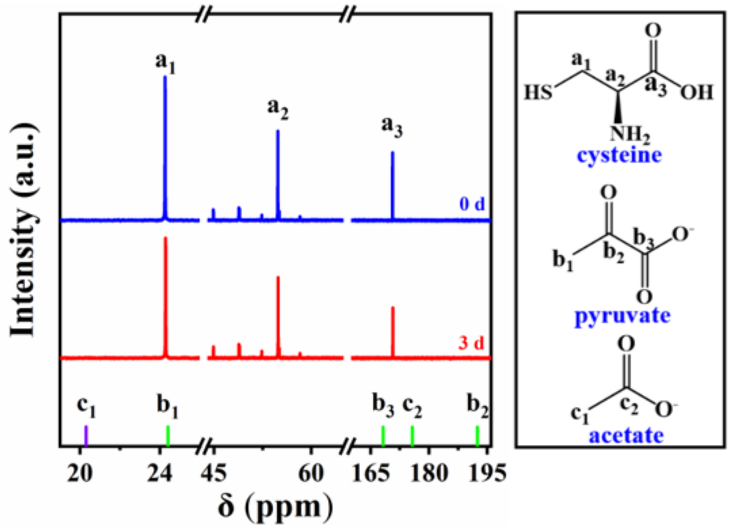

Supplementary Fig. 12 ^{12}C NMR spectra of metabolic intermediates by *M. b* with cysteine as the potential carbon source for 3 days. Source data are provided as a Source Data file.

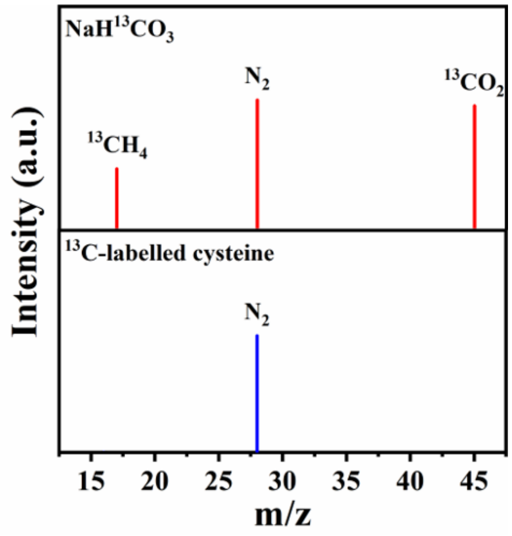

Supplementary Fig. 13 Mass spectrometric analysis of headspace gas with ^{13}C-labelled $NaHCO_3$ or cysteine as the carbon source for 3 days, using *M. b*-NiCu@CdS as catalyst. Source data are provided as a Source Data file.

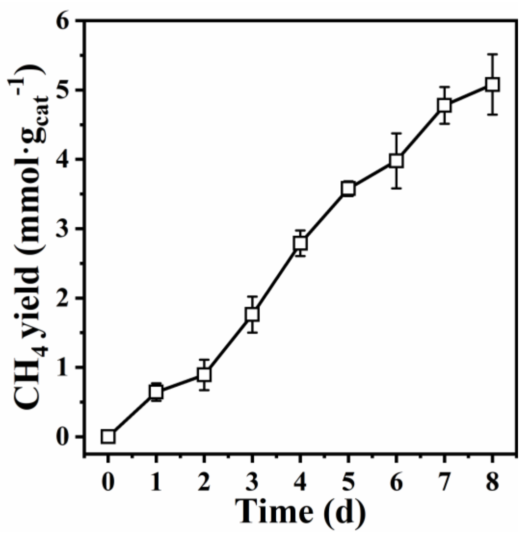

Supplementary Fig. 14 CH_4 yield in *M. b*-NiCu@CdS hybrid system using KI as the sacrificial reducing agent. The measurements are performed in 50 mL of sterilized autotrophic medium with CO_2 as the only carbon source under irradiation of 395 ± 5 nm LEDs (0.8 ± 0.1 mW cm^{-2}). Data are presented as mean values ± standard deviation derived from n = 3 independent experiments. Source data are provided as a Source Data file.

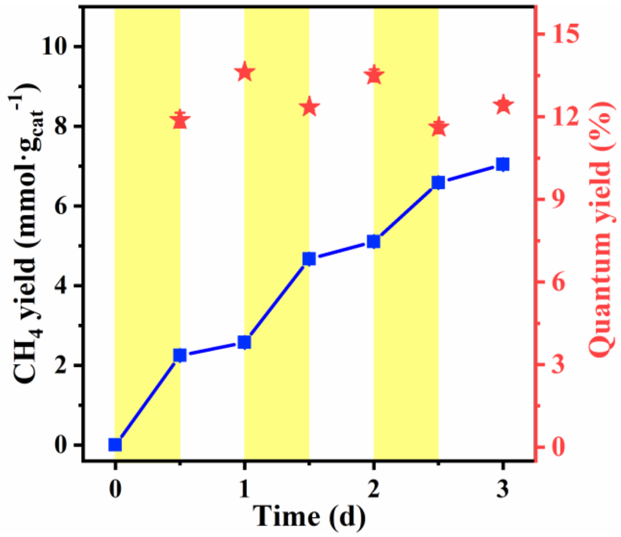

Supplementary Fig. 15 CH_4 yield and quantum yield with *M. b*-NiCu@CdS in light-dark cycles. Data are presented as mean values ± standard deviation derived from n = 3 independent experiments. Source data are provided as a Source Data file.

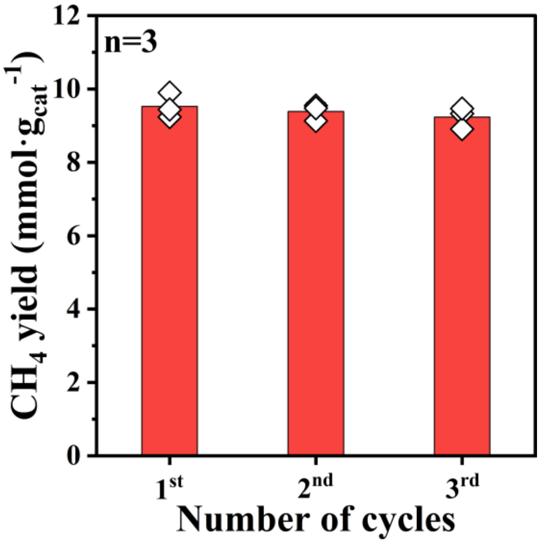

Supplementary Fig. 16 CH_4 yield with *M. b*-NiCu@CdS for 3 cycles of operation, each of which lasts 5 days. Data are presented as mean values ± standard deviation derived from n = 3 independent experiments. Source data are provided as a Source Data file.

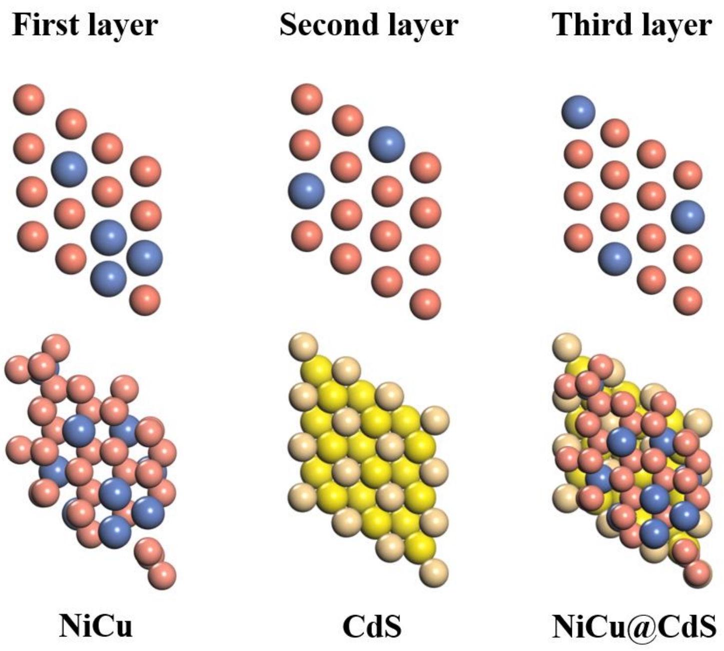

Supplementary Fig. 17 The detailed schematic illustration of NiCu@CdS model used for DFT calculation. Dark blue, pink, light brown and yellow balls represent Ni, Cu, Cd and S atoms, respectively.

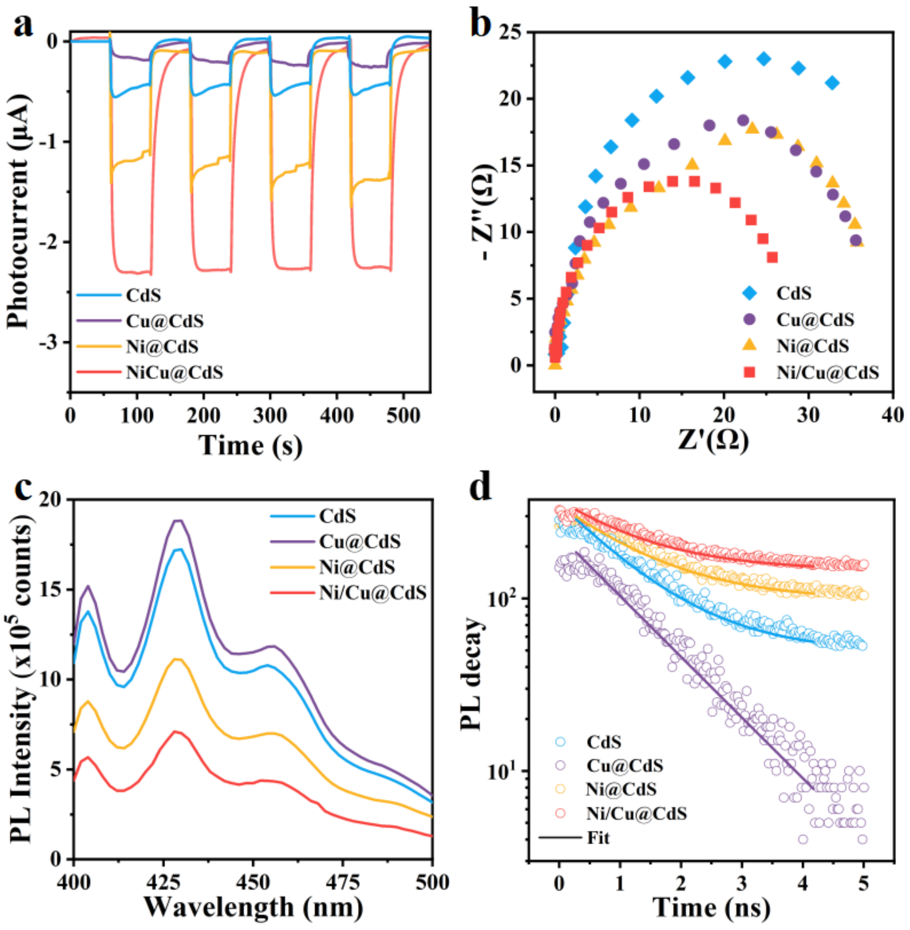

Supplementary Fig. 18 Photoelectrochemical characterization. (a) *I*–*t* curves of NiCu@CdS in comparison with those of Ni@CdS, Cu@CdS and CdS under UV light irradiation. (b) Electrochemical impedance spectroscopy (EIS) Nyquist plots of NiCu@CdS in reference to Ni@CdS, Cu@CdS and CdS. (c) PL spectra and (d) PL decay curves of NiCu@CdS in reference to Ni@CdS, Cu@CdS and CdS. *I*-*t* experiments (applied potential: –300 mV versus saturated calomel electrode (SCE); light on/off cycle: 60 s; light source: 395 ± 5 nm ultraviolet LEDs) and EIS experiments (applied potentials: an open-circuit potential; frequency range: 1000 kHz to 0.01 Hz; sinusoidal perturbation amplitude: 5 mV) are evaluated in a three-electrode anaerobic electrochemical quartz chamber connected to a CHI 660E electrochemical station (CH instruments, TX, USA). Working electrode: indium tin oxide (ITO)-coated glass plate (radius: 1.5 cm) placed at the bottom of the chamber with 6.48 mg of self-precipitated samples; counter electrode: a platinum sheet (1 × 1 cm); reference electrode: SCE in the salt bridge filled with saturated KCl; electrolyte: autotrophic medium. Source data are provided as a Source Data file.

The photoelectrochemical characteristics of different CdS-based materials are measured. A significant photocurrent improvement up to 2.31 μA through the coupling of CdS and NiCu alloys is observed (Supplementary Fig. 18a), indicating that the NiCu alloy enhances electron transfer

and utilization. This finding is verified by electrochemical impedance spectroscopy (EIS). The Nyquist plots demonstrate that the introduction of NiCu alloy significantly reduces the charge transfer resistance (indicated by a smaller diameter of the semicircle at high frequencies) (Supplementary Fig. 18b). The charge separation of photocatalysts is highly important to photocatalytic CO_2 conversion and can be evaluated by the photoluminescence (PL) intensity and lifetime. Steady-state PL spectra are shown in Supplementary Fig. 18c, in which a significantly weaker fluorescence is evident after the introduction of NiCu alloy, demonstrating that the recombination of photogenerated charges has been significantly suppressed. In particular, Supplementary Fig. 18d shows the representative time-resolved PL decays after pulsed excitation at λ = 380 nm. Curve fitting with an exponential model (Supplementary Table 6) shows lifetimes of 1.30, 1.27, 1.47 and 2.04 ns for the prepared CdS, Cu@CdS, Ni@CdS and NiCu@CdS, respectively, indicating the facilitated charge separation by the NiCu alloy.

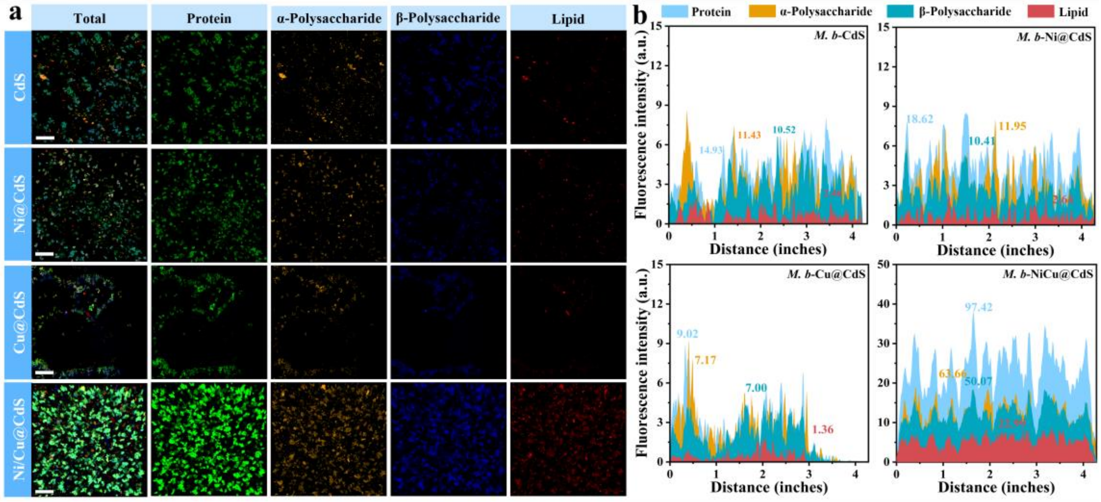

Supplementary Fig. 19 Component and concentration analysis of EPS. (a) Fluorescence staining of EPS in *M. b*-CdS-based hybrid systems after 3-day irradiation of 395 ± 5 nm LEDs (0.8 ± 0.1 mW cm^{-2}), and (b) the corresponding fluorescence intensity curves of protein (green), α-polysaccharide (orange), β-polysaccharide (blue) and lipid (red). The values of the light colour are the corresponding integrals of fluorescence intensities of different components. Scale bars: 20 μm in a. Source data are provided as a Source Data file.

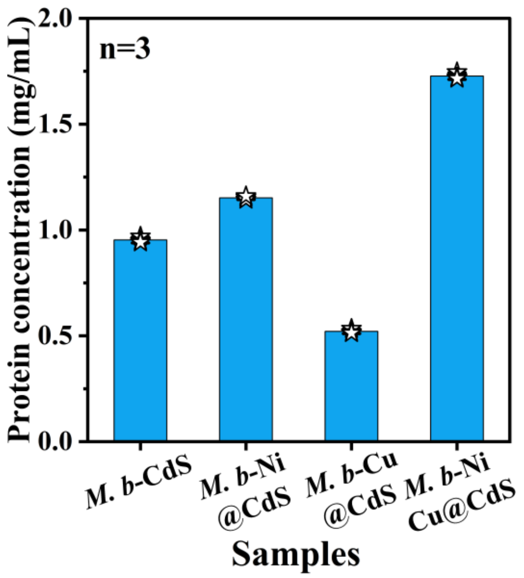

Supplementary Fig. 20 Concentration of membrane-bound proteins in *M. b*-CdS-based hybrid systems after 3-day irradiation of 395 ± 5 nm LEDs (0.8 ± 0.1 mW cm^{-2}). Data are presented as mean values ± standard deviation derived from n = 3 independent experiments. Source data are provided as a Source Data file.

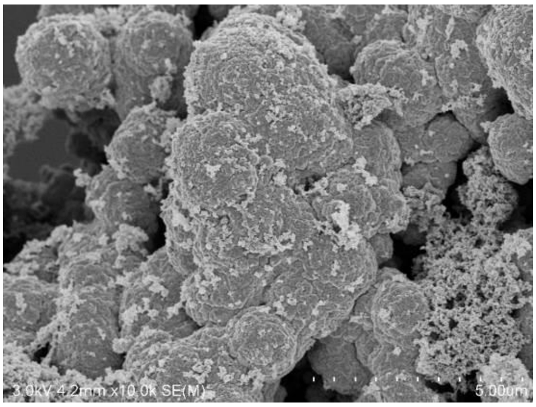

Supplementary Fig. 21 Scanning electron microscopy (SEM) image of *M. b*-NiCu@CdS after three 5-day cycles of light irradiation.

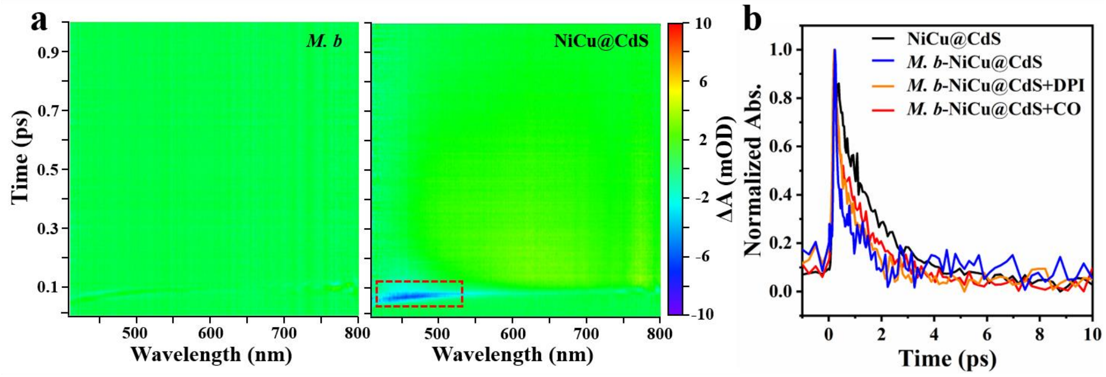

Supplementary Fig. 22 Transient absorption (TA) spectroscopy and the electron recombination kinetics. (a) Transient absorption spectroscopy excited at 365 nm (The red rectangle shows the transient photoinduced absorption band). (b) Comparison of decay kinetics of NiCu@CdS only (cell-free), *M. b*-NiCu@CdS, *M. b*-NiCu@CdS+CO (H_2ase inhibition), and *M. b*-NiCu@CdS+DPI (cytochrome inhibition). Source data are provided as a Source Data file.

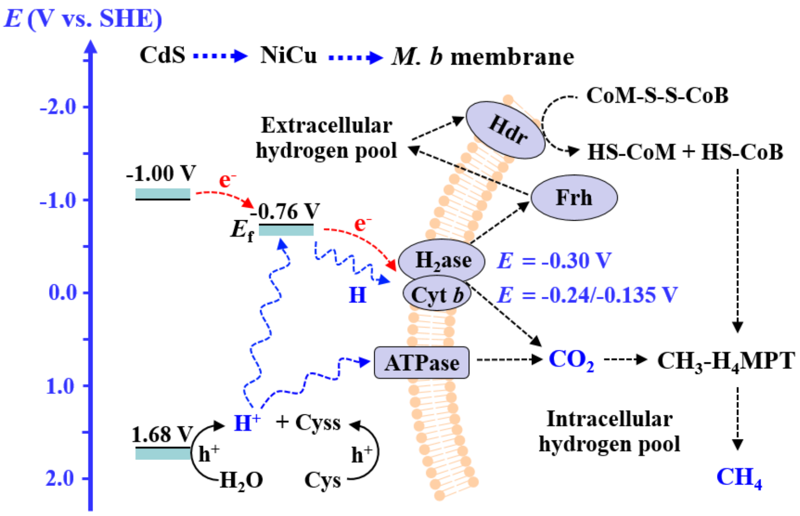

Supplementary Fig. 23 The full energy level diagram with *M. b*-NiCu@CdS hybrid.

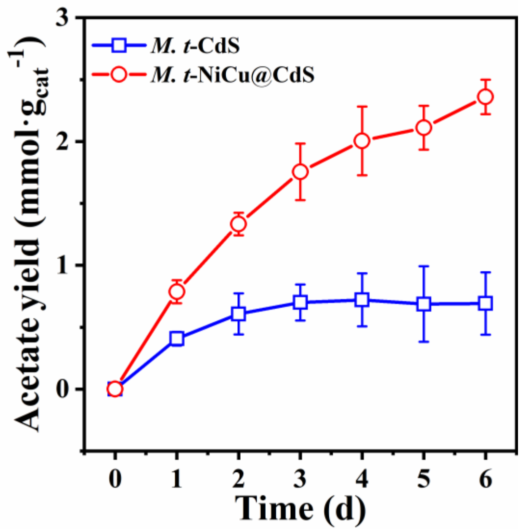

Supplementary Fig. 24 Acetate yields in *Moorella thermoacetica*-CdS-based hybrid systems. The measurements are performed in 50 mL of sterilized autotrophic medium under irradiation of 395 ± 5 nm LEDs (0.8 ± 0.1 mW cm^{-2}). Data are presented as mean values ± standard deviation derived from n = 3 independent experiments. Source data are provided as a Source Data file.

Supplementary Table 1 Structural and compositional properties of the NiCu alloy.

Sample	Metal content (wt%)[a]		Ni:Cu (atomic ratio)		
	Ni	Cu	Theo.[b]	XPS[c]	EDX-TEM[d]
M. b-50%Ni@CdS	28.7	0	–	–	–
M. b-50%Ni_2Cu_8@CdS	5.23	23.4	0.25	0.28	0.20
M. b-50%Ni_5Cu_5@CdS	13.83	14.89	1.00	0.94	0.92
M. b-50%Cu@CdS	0	27.62	–	–	–

[a] Theoretical Ni and Cu contents.

[b] Theoretical Ni:Cu atomic ratio.

[c] Ni:Cu atomic ratio estimated from the XPS spectra.

[d] Ni:Cu atomic ratio estimated from the EDS collected in TEM mode.

Supplementary Table 2 Fitting results of EXAFS data.

	Path	CN[a]	*R* (Å)[b]	σ^2 (Å^2)[c]	ΔE_0 (eV)[d]	*R* factor
Ni foil	Ni–Ni	12.00	2.48	0.006	6.82	0.002
Ni-*K* edge	Ni–Ni/Cu	9.08	2.49	0.006	-3.51	0.016
Cu foil	Cu–Cu	12.00	2.54	0.008	4.04	0.003
Cu-*K* edge	Cu–Cu/Ni	8.63	2.51	0.016	1.83	0.011

[a] CN: coordination number; [b] *R*: bond length; [c] σ^2: Debye-Waller factor; [d] ΔE_0: the inner potential correction. *R* factor: goodness of fit. S_0^2 is set as 0.814 (Ni) and 0.844 (Cu) for Ni–Ni/Cu–Cu, which is obtained from the experimental EXAFS of Ni/Cu foil references by fixing CN as the known crystallographic value and is fixed to all the samples.

* *K*-range = 3.0–11.0 Å, *R*-range = 1.0–3.0 Å

Supplementary Table 3 Production yield and product selectivity for the photocatalytic conversion of CO_2 over biotic-abiotic hybrid systems.

Biotic-abiotic systems	Average production yield (μmol g_{cat}^{-1} h^{-1})		Product selectivity for CH_4 (%) [a]
	CH_4	H_2	
M. b-CdS	15.54	14.97	50.93
M. b-Ni@CdS	21.13	7.86	72.89
M. b-Cu@CdS	8.14	23.29	25.90
M. b-NiCu@CdS	79.38	0.77	99.04
NiCu@CdS+*M. b*	42.98	319.70	11.85

[a] The product selectivity for CH_4 production is evaluated based on the amounts of products using the following equation: Product selectivity (%) = $[v(CH_4)] / [v(CH_4) + v(H_2)] \times 100\%$, where $v(H_2)$ and $v(CH_4)$ stand for the formation rates for H_2 and CH_4. No other products have been observed in the biotic-abiotic hybrid system.

Supplementary Table 4 Comparison of quantum yields for photocatalytic reactions in different biotic-abiotic hybrid systems.

Model bacteria	Semiconductors	Products	Light intensity ($mWcm^{-2}$)	Quantum yield (%)	Reference
Methanosarcina barkeri	Ni/Cu@CdS	CH_4	0.80	12.41 ± 0.16	This work
Methanosarcina barkeri	Ni:CdS	CH_4	0.80	2.08 ± 0.03	[17]
Methanosarcina barkeri	CdS	CH_4	1.00	0.34	[1]
Rhodopseudomonas palustris	CdS	NH_3	3.50	3.30 ±0.8	[18]
Rhodopseudomonas palustris	CdS	NH_3	10.00	6.73	[19]
Rhodopseudomonas palustris	CdS	C_2^+	8.00	5.98	[20]
Escherichia coli	CdS	H_2	0.04	4.00	[21]
Escherichia coli	CdS	H_2	200.00	7.93	[22]
Escherichia coli	GaN:ZnO	H_2	3.40	1.88	[23]
Moorella thermoacetica	CdS	Acetate	2.00	2.44 ± 0.62	[24]
Moorella thermoacetica	PDI/PFP	Acetate	5.00	1.60	[25]
Moorella thermoacetica	$Au_{22}(SG)_{18}$	Acetate	2.00	2.86 ± 0.38	[26]
Thiobacillus denitrificans	CdS	NO_2^-	3.07	2.00 ±0.2	[27]
Saccharomyces cerevisiae	InP	Shikimic acid	5.60	1.58 ± 0.05	[28]
Plants			100.00	< 1	[29]

Supplementary Table 5 Comparison of CH_4 production yield and electron selectivity for the photocatalytic conversion of CO_2 in different material systems.

Materials	Products	Average CH_4 production yield (μmol g_{cat}^{-1} h^{-1})	Electron selectivity for CH_4 (%)[a]	Light intensity (mW cm^{-2})	Quantum yield (%)	Reference
M. b-Ni/Cu @CdS	CH_4	79.38	~100	0.80	12.41 ± 0.16	This work
$CuIn_5S_8$	CH_4	8.7	~100	50.00	0.79	[30]
Pd_7Cu_1-TiO_2	CH_4, CO, H_2	19.6	96	2.00	–	[31]
Ni:CdS	CH_4, CO	~9.5	~85	100.00	–	[32]
SiC/RGO	CH_4, CO	6.72	92	860.00	–	[33]
$CsPbBr_3$/GO	CH_4, CO, H_2	23.7	99.3% for CO and CH_4	6.25	~0.025	[34]
$Fe_2V_4O_{13}$/RGO/ CdS	CH_4, O_2	~2.04	–	–	–	[35]
$SrNb_2O_6$-plate-105	CH_4, CO, H_2	19.9	82% for CO and CH_4	100.00	0.07	[36]
TiO_2	CH_4, CO	0.94	17.8	20.00	1.32	[37]
meso-TiO_2@$ZnIn_2S_4$/Ti_3C_2 MXene	CH_4, CO	11.33	52.7	100.00	–	[38]
CQD/OCN-25 %	CH_4	0.15	70	–	–	[39]
Bi_2WO_6-Cl_3	CH_4	1.66	94.98	–	0.08	[40]
$W_{18}O_{49}$/Cu_2O	CH_4	17.2	94.7	80.00	–	[41]
Silver	CH_4	19.31	~100	–	–	[42]
2Au-CN	CH_4	1.55	19.1	–	–	[43]
TiO_{2-x}	CH_4	1.63	85.8	100	0.038	[44]
Au/TiO_2/$W_{18}O_4$	CH_4, CO	35.55	93.3	50	0.02	[45]

[a] The electron selectivity for CH_4 production is evaluated based on the required electrons using the following equation: Electron selectivity (%) = $[8v(CH_4)]/[8v(CH_4) + 2v(H_2)] \times 100\%$, where $v(H_2)$ and $v(CH_4)$ stand for the formation rates for H_2 and CH_4.

Supplementary Table 6 Fitting parameters for PL decay curves of CdS-based materials.

Materials	A	τ (ns)
CdS	242.12	1.30
Cu@CdS	228.42	1.27
Ni@CdS	188.18	1.47
NiCu@CdS	262.35	2.04

Supplementary Table 7 Biexponential fitting data for TA spectra.

Systems	τ_1 (ps)	τ_2 (ps)
NiCu@CdS	0.41 ± 0.06	1.51 ± 0.12
M. b-NiCu@CdS	0.05 ± 0.01	0.22 ± 0.05
M. b-NiCu@CdS+CO	0.77 ± 0.06	0.13 ± 0.03
M. b-NiCu@CdS+DPI	0.04 ± 0.01	0.75 ± 0.01

Supplementary Table 8 The composition of contained substrate medium (heterotrophic medium) and uncontained substrate medium (autotrophic medium) for *Methanosarcina barkeri*.

Component	Heterotrophic (g L^{-1})	Autotrophic (g L^{-1})
$MgCl_2 \cdot 6H_2O$	0.4	0.4
$CaCl_2 \cdot 2H_2O$	0.1	0.1
NH_4Cl	0.1	0.1
KH_2PO_4	0.2	0.2
KCl	0.5	0.5
HEPES	7.16	7.16
$NaHCO_3$	2.52	2.52
$Na_2S \cdot 9H_2O$	0.24	–
NaAc	1.394	–
Cysteine-HCl	–	2.0
Trace element solution	1 mL	1 mL
ST solution	1 mL	1 mL
Vitamin solution	3 mL VS	3 mL VS

Trace element solution refers to Supplementary Table 7.2. ST solution refers to Supplementary Table 7.3. Vitamin solution refers to Supplementary Table 7.4.

Supplementary Table 8.2 Trace element solution (per liter, the medium containing) that refers to Supplementary Table 8.

Component	Content
HCl (2 M)	50 mL
$FeCl_2 \cdot 4H_2O$	2 g
$ZnCl_2$	0.20 g
$MnCl_2 \cdot 4H_2O$	0.10 g
H_3BO_3	0.18 g
$CoCl_2 \cdot 6H_2O$	0.05 g
$CuCl_2 \cdot 2H_2O$	6 mg
$NiCl_2 \cdot 6H_2O$	72 mg
$Na_2MoO_4 \cdot 2H_2O$	108 mg

Supplementary Table 8.3 ST solution (per liter, the medium containing) that refers to Supplementary Table 8.

Component	Content
NaOH	0.5 g
$Na_2SeO_3 \cdot 5H_2O$	3 mg
$Na_2WO_4 \cdot 2H_2O$	4 mg

Supplementary Table 8.4 Vitamin solution (VS, per liter, the medium containing) that refers to Supplementary Table 8.

Component	Content
4-aminobenzoic acid	0.04 g
DL-a-lipoic acid	0.01 g
Calcium-D(+)-panto-thenate	0.10 g
Pyridoxine-HCl	0.10 g
Folic acid	0.03 g
Nicotinic acid	0.05 g
Riboflavin	0.05 g
Thiamin-HCl $\cdot 2H_2O$	0.01 g
Vitamin B_{12}	0.05 g

Supplementary Table 9 The composition of contained substrate medium (heterotrophic medium) and uncontained substrate medium (autotrophic medium) for *Moorella thermoacetica*.

Component	Heterotrophic (g L^{-1})	Autotrophic (g L^{-1})
NaCl	0.40	0.40
NH_4Cl	0.40	0.40
$MgSO_4 \cdot 7H_2O$	0.33	0.33
$CaCl_2$	0.05	0.05
KCl	0.25	0.25
Yeast Extract	0.50	–
Tryptone	0.50	–
$NaHCO_3$	2.50	2.50
KH_2PO_4	–	0.64
β-glycerophosphate $2Na \cdot xH_2O$	0.80	–
Trace Mineral Mix	10 mL	10 mL
Wolfe's Vitamin Mix	10 mL	10 mL

Trace Mineral Mix refers to Supplementary Table 9.2. Wolfe's Vitamin Mix refers to Supplementary Table 9.3.

Supplementary Table 9.2 Trace Mineral Mix (per liter, the medium containing) that refers to Supplementary Table 9.

Component	Content
Nitriloacetic acid	2.0 g
$MnSO_4 \cdot H_2O$	1.0 g
$Fe(SO_4)_2(NH_4)_2 \cdot 6H_2O$	0.80 g
$CoCl_2 \cdot 6H_2O$	0.20 g
$ZnSO_4 \cdot 7H_2O$	0.20 mg
$CuCl_2 \cdot 2H_2O$	20 mg
$NiCl_2 \cdot 6H_2O$	20 mg
$Na_2MoO_4 \cdot 2H_2O$	20 mg
Na_2SeO_4	20 mg
Na_2WO_4	20 mg

Supplementary Table 9.3 Wolfe's Vitamin Mix (per liter, the medium containing) that refers to Supplementary Table 9.

Component	Content
Pyridoxine-HCl	10 mg
Thiamin-HCl	5.0 mg
Riboflavin	5.0 mg
Nicotinic acid	5.0 mg
Calcium-D(+)-panto-thenate	5.0 mg
Thioctic acid	5.0 mg
Biotin	2.0 mg
Folic acid	2.0 mg
Vitamin B_{12}	0.10 mg

References

1. Ye, J. et al. Light-driven carbon dioxide reduction to methane by *Methanosarcina barkeri*-CdS biohybrid. *Appl. Catal. B Environ.* **257**, 117916 (2019).
2. Hook, S. E., Steele, M. A., Northwood, K. S., Wright, A. D. G. & McBride, B. W. Impact of high-concentrate feeding and low ruminal pH on methanogens and protozoa in the rumen of dairy cows. *Microb. Ecol.* **62**, 94–105 (2011).
3. Staley, B. F., de los Reyes, F. L. & Barlaz, M. A. Effect of spatial differences in microbial activity, pH, and substrate levels on methanogenesis initiation in refuse. *Appl. Environ. Microbiol.* **77**, 2381–2391 (2011).
4. Garcia, J. L., Patel, B. K. C. & Ollivier, B. Taxonomic, phylogenetic, and ecological diversity of methanogenic *Archaea*. *Anaerobe* **6**, 205–226 (2000).
5. Waiskopf, N. et al. Photocatalytic reactive oxygen species formation by semiconductor-metal hybrid nanoparticles. Toward light-induced modulation of biological processes. *Nano Lett.* **16**, 4266–4273 (2016).
6. Xiao, K. et al. Photocatalytic bacterial inactivation by a rape pollen-MoS_2 biohybrid catalyst: Synergetic effects and inactivation mechanisms. *Environ. Sci. Technol.* **54**, 537-549 (2020).
7. Vaquero, F., Navarro, R. M. & Fierro, J. L. G. Influence of the solvent on the structure, morphology and performance for H_2 evolution of CdS photocatalysts prepared by solvothermal method. *Appl. Catal. B Environ.* **203**, 753–767 (2017).
8. Di, T., Zhu, B., Zhang, J., Cheng, B. & Yu, J. Enhanced photocatalytic H_2 production on CdS nanorod using cobalt-phosphate as oxidation cocatalyst. *Appl. Surf. Sci.* **389**, 775–782 (2016).
9. Yu, J., Yu, Y., Zhou, P., Xiao, W. & Cheng, B. Morphology-dependent photocatalytic H_2-production activity of CdS. *Appl. Catal. B Environ.* **156**–**157**, 184–191 (2014).
10. Ahsan, M. A. et al. Tuning of trifunctional NiCu bimetallic nanoparticles confined in a porous carbon network with surface composition and local structural distortions for the electrocatalytic oxygen reduction, oxygen and hydrogen evolution reactions. *J. Am. Chem. Soc.* **142**, 14688–14701 (2020).
11. Jin, Z. & Zhang, L. Performance of Ni-Cu bimetallic co-catalyst g-C_3N_4 nanosheets for improving hydrogen evolution. *J. Mater. Sci. Technol.* **49**, 144–156 (2020).
12. Huang, Z. et al. Shape-controlled synthesis of CuS as a Fenton-like photocatalyst with high catalytic performance and stability. *J. Alloys Compd.* **896**, 163045 (2022).
13. Zhao, B. et al. Constructing hierarchical hollow CuS microspheres: Via a galvanic replacement reaction and their use as wide-band microwave absorbers. *CrystEngComm* **19**, 2178–2186 (2017).
14. Hong, J. et al. Room temperature wafer-scale synthesis of highly transparent, conductive CuS nanosheet films via a simple sulfur adsorption-corrosion method. *ACS Appl. Mater. Interfaces* **13**, 4244–4252 (2021).
15. Liu, H. et al. Performance and mechanism of CuS-modified MWCNTs on mercury removal: Experimental and density functional theory study. *Fuel* **309**, 122238 (2022).
16. Dai, X. et al. CuS/$KTa_{0.75}Nb_{0.25}O_3$ nanocomposite utilizing solar and mechanical energy for catalytic N_2 fixation. *J. Colloid Interface Sci.* **603**, 220–232 (2021).
17. Ye, J. et al. Efficient photoelectron capture by Ni decoration in *Methanosarcina barkeri*-CdS biohybrids for enhanced photocatalytic CO_2-to-CH_4 conversion. *iScience* **23**, 101287 (2020).
18. Brown, K. A. et al. Light-driven dinitrogen reduction catalyzed by a CdS:nitrogenase MoFe

protein biohybrid. *Science* **352**, 448–450 (2016).
19. Wang, B. et al. Biohybrid photoheterotrophic metabolism for significant enhancement of biological nitrogen fixation in pure microbial cultures. *Energy Environ. Sci.* **12**, 2185–2191 (2019).
20. Wang, B., Jiang, Z., Yu, J. C., Wang, J. & Wong, P. K. Enhanced CO_2 reduction and valuable C_2^+ chemical production by a CdS-photosynthetic hybrid system. *Nanoscale* **11**, 9296–9301 (2019).
21. Martins, M., Toste, C. & Pereira, I. A. C. Enhanced light-driven hydrogen production by self-photosensitized biohybrid systems. *Angew. Chem. Int. Ed.* **60**, 9055–9062 (2021).
22. Wang, B. et al. Enhanced biological hydrogen production from *Escherichia coli* with surface precipitated cadmium sulfide nanoparticles. *Adv. Energy Mater.* **7**, 1–10 (2017).
23. Kosem, N. et al. Photobiocatalytic H_2 evolution of GaN:ZnO and [FeFe]-hydrogenase recombinant *Escherichia coli*. *Catal. Sci. Technol.* **10**, 4042–4052 (2020).
24. Sakimoto, K. K., Wong, A. B. & Yang, P. Self-photosensitization of nonphotosynthetic bacteria for solar-to-chemical production. *Science* **351**, 74–77 (2016).
25. Gai, P. et al. Solar-powered organic semiconductor–bacteria biohybrids for CO_2 reduction into acetic acid. *Angew. Chem. Int. Ed.* **59**, 7224–7229 (2020).
26. Zhang, H. et al. Bacteria photosensitized by intracellular gold nanoclusters for solar fuel production. *Nat. Nanotechnol.* **13**, 900–905 (2018).
27. Chen, M. et al. Light-driven nitrous oxide production via autotrophic denitrification by self-photosensitized *Thiobacillus denitrificans*. *Environ. Int.* **127**, 353–360 (2019).
28. Guo, J. et al. Light-driven fine chemical production in yeast biohybrids. *Science* **362**, 813–816 (2018).
29. Liu C., Colón, B. C., Ziesack, M., Silver, P. A. & Nocera, D. G. Water splitting–biosynthetic system with CO_2 reduction efficiencies exceeding photosynthesis. *Science* **352**, 1210-1213 (2016).
30. Li, X. et al. Selective visible-light-driven photocatalytic CO_2 reduction to CH_4 mediated by atomically thin $CuIn_5S_8$ layers. *Nat. Energy* **4**, 690–699 (2019).
31. Long, R. et al. Isolation of Cu atoms in Pd lattice: Forming highly selective sites for photocatalytic conversion of CO_2 to CH_4. *J. Am. Chem. Soc.* **139**, 4486–4492 (2017).
32. Wang, J. et al. Enabling visible-light-driven selective CO_2 reduction by doping quantum dots: Trapping electrons and suppressing H_2 evolution. *Angew. Chem. Int. Ed.* **57**, 16447–16451 (2018).
33. Han, C., Lei, Y., Wang, B. & Wang, Y. In situ-fabricated 2D/2D heterojunctions of ultrathin SiC/reduced graphene oxide nanosheets for efficient CO_2 photoreduction with high CH_4 selectivity. *ChemSusChem* **11**, 4237–4245 (2018).
34. Xu, Y. F. et al. A $CsPbBr_3$ perovskite quantum dot/graphene oxide composite for photocatalytic CO_2 reduction. *J. Am. Chem. Soc.* **139**, 5660–5663 (2017).
35. Li, P. et al. All-solid-state Z-scheme system arrays of $Fe_2V_4O_{13}$/RGO/CdS for visible light-driving photocatalytic CO_2 reduction into renewable hydrocarbon fuel. *Chem. Commun.* **51**, 800–803 (2015).
36. Xie, S., Wang, Y., Zhang, Q., Deng, W. & Wang, Y. $SrNb_2O_6$ nanoplates as efficient photocatalysts for the preferential reduction of CO_2 in the presence of H_2O. *Chem. Commun.* **51**, 3430–3433 (2015).
37. Fang, B. et al. Large-scale synthesis of TiO_2 microspheres with hierarchical nanostructure for highly efficient photodriven reduction of CO_2 to CH_4. *ACS Appl. Mater. Interfaces* **6**, 15488–15498 (2014).

38. Wang, K. et al. Z-scheme core-shellmeso-TiO_2@$ZnIn_2S_4$/Ti_3C_2MXene enhances visible light-driven CO_2-to-CH_4 selectivity. *Ind. Eng. Chem. Res.* **60**, 8720–8732 (2021).
39. Li, Q. et al. Enhanced CH_4 selectivity in CO_2 photocatalytic reduction over carbon quantum dots decorated and oxygen doping g-C_3N_4. *Nano Res.* **12**, 2749–2759 (2019).
40. Li, Y. Y. et al. Selective photocatalytic reduction of CO_2 to CH_4 modulated by chloride modification on Bi_2WO_6 nanosheets. *ACS Appl. Mater. Interfaces* **12**, 54507–54516 (2020).
41. Jiang, M. et al. Tuning $W_{18}O_{49}$/Cu_2O {111} Interfaces for the highly selective CO_2 photocatalytic conversion to CH_4. *ACS Appl. Mater. Interfaces* **12**, 35113–35119 (2020).
42. Fu, Z. et al. Controllable synthesis of porous silver cyanamide nanocrystals with tunable morphologies for selective photocatalytic CO_2 reduction into CH_4. *J. Colloid Interface Sci.* **593**, 152–161 (2021).
43. Li, H., Gao, Y., Xiong, Z., Liao, C. & Shih, K. Enhanced selective photocatalytic reduction of CO_2 to CH_4 over plasmonic Au modified g-C_3N_4 photocatalyst under UV–vis light irradiation. *Appl. Surf. Sci.* **439**, 552–559 (2018).
44. Xing, M. et al. Modulation of the reduction potential of TiO_{2-x} by fluorination for efficient and selective CH_4 generation from CO_2 photoreduction. *Nano Lett.* **18**, 3384–3390 (2018).
45. Jiang, X. et al. Plasmonic active “Hot Spots”-confined photocatalytic CO_2 reduction with high selectivity for CH_4 production. *Adv. Mater.* **34**, 2109330 (2022).
